# PI3K/AKT Signal Pathway: A Target of Natural Products in the Prevention and Treatment of Alzheimer’s Disease and Parkinson’s Disease

**DOI:** 10.3389/fphar.2021.648636

**Published:** 2021-04-15

**Authors:** Hui-Zhi Long, Yan Cheng, Zi-Wei Zhou, Hong-Yu Luo, Dan-Dan Wen, Li-Chen Gao

**Affiliations:** ^1^ Department of Pharmacy, Cancer Institute, Phase I Clinical Trial Centre, Changsha Central Hospital Affiliated to University of South China, School of Pharmacy, University of South China, Changsha, China; ^2^ Hunan Provincial Key Laboratory of Tumor Microenvironment Responsive Drug Research, Hengyang, China

**Keywords:** PI3K/Akt signal pathway, neurodegenerative diseases, Alzheimer’s disease, Parkinson’s disease, natural products, nerve protection

## Abstract

Alzheimer’s disease (AD) and Parkinson’s disease (PD) are two typical neurodegenerative diseases that increased with aging. With the emergence of aging population, the health problem and economic burden caused by the two diseases also increase. Phosphatidylinositol 3-kinases/protein kinase B (PI3K/AKT) signaling pathway regulates signal transduction and biological processes such as cell proliferation, apoptosis and metabolism. According to reports, it regulates neurotoxicity and mediates the survival of neurons through different substrates such as forkhead box protein Os (FoxOs), glycogen synthase kinase-3β (GSK-3β), and caspase-9. Accumulating evidences indicate that some natural products can play a neuroprotective role by activating PI3K/AKT pathway, providing an effective resource for the discovery of potential therapeutic drugs. This article reviews the relationship between AKT signaling pathway and AD and PD, and discusses the potential natural products based on the PI3K/AKT signaling pathway to treat two diseases in recent years, hoping to provide guidance and reference for this field. Further development of Chinese herbal medicine is needed to treat these two diseases.

## Introduction

Neurodegenerative diseases are a kind of diseases that gradually lose the structure or function of neurons, including Parkinson’s disease (PD), Alzheimer’s disease (AD) and Amyotrophic lateral sclerosis (ALS) and so on. These diseases are characterized by progressive degeneration and neuronal necrosis, resulting in cognitive, emotional and behavioral abnormalities (Hetman et al., 2020). With the acceleration of population ages, the number of people suffering from AD and PD is increasing year by year. Up to now, the current treatment strategies of these diseases aim to alleviate symptoms and/or inhibit disease progression without radical cure, posing a serious threat to the quality of patients’ life, hence becoming a global health problem. The high cost of treatment and nursing not only brings great trouble to patients and their families, but also causes great social and economic burden (Batista and Pereira, 2016), making the development of this kind of new drugs an urgent problem in the field of medicine. In addition, abnormal injuries caused by chemotherapy drugs give rise to different degrees of impact on patients’ physical and mental health and quality of life, which have become the direct cause of dosage restriction or discontinuation of some drugs (Vaz and Silvestre, 2020). A systematic review showed that Chinese herbal medicine and its natural active ingredients are of good safety and tolerance in protecting the central nervous system injury, and so that it gets increasing attention by the medical community (Yang et al., 2017).

Phosphatidylinositol 3-kinase/protein kinase B (PI3K/AKT) signal pathway has been proved to play an important role in the central nervous system (Matsuda et al., 2019). It has been diffusely studied for its involvement in the physiological processes of central nervous system, such as cell survival, autophagy, neurogenesis, neuronal proliferation and differentiation, and synaptic plasticity. In recent years, a growing number of studies have found that many natural products based on PI3K/AKT signal pathway protect dopaminergic neurons, hippocampal neurons, cortical neurons, and inhibit the activation of microglia, thus playing a role in the prevention and treatment of AD and PD. In this paper, we obtain relevant literature through Pubmed, Web of Science, China National Knowledge Infrastructure (CNKI) and other extensive databases. And we systematically summarize the related studies of natural products for the prevention and treatment of AD and PD based on PI3K/AKT signal pathway.

## PI3K/AKT Signal Pathway: Overview

### PI3K: The Key Component Upstream of the Pathway

PI3Ks, a family of intracellular lipid kinases, are key elements in the upstream of the PI3K/AKT signaling pathway. PI3Ks are divided into three classes (I-III) according to their structure and substrate selectivity. The most generally studied class I isoforms that are activated by cell surface receptors are heterodimers composed of a regulatory subunit (P85) and a catalytic subunit (P110). The amino terminus of P85 contains a Src Homology 3 (SH3) domain and two proline-rich regions, while the basal terminus contains two SH2 domains and a non-coding region that combine with P110. Besides, class I isoforms are further segmented into class IA (PI3Kα, β and δ) and class IB (PI3Kγ) based on their modes of regulation (Thorpe et al., 2015). Class IA consists of catalytic subunits p110α, β, δ and regulatory subunits p85α, β, *γ*. Class IB are constituted by a p110γ catalytic subunit coupled with the regulatory isoforms p101 or p87. The differences lie in the way of the activation between the two kinases. PI3Kα, β and δ are activated when extracellular ligands bind to a transmembrane glycoprotein receptor tyrosine kinase (RTK) with enzyme activity, while PI3Kγ is activated by G protein coupled receptors (GPCRs) and Ras family GTP enzymes (Dobbin and Landen, 2013). There are three class II isoforms, PI3KC2α, 2β, 2γ, which may constitutively bind to membranes and require additional activation signals. And the single class III PI3K, vacuolar protein sorting 34 (VPS34), is significant for membrane traffic from the plasma membrane to early endosomes (Sugiyama et al., 2019).

### AKT: The Core Site of the Pathway

Protein kinase B (PKB), a serine/threonine kinase, is considered as one of the most important effector kinase downstream of PI3K and the core of PI3K/AKT signal pathway. Different genes encode three highly homologous subtypes of AKT: AKT1/PKBα, AKT2/PKBβ, and AKT3/PKBγ. Each isoform contains a conserved N-terminal plekstrin homology (PH) domain, a central fragment, and a C-terminal regulatory domain (Hemmings et al., 2004). Membrane translocation in AKT activation is mediated by the PH domain, and AKT activity is weakened due to its mutation or deletion. The structure of the catalytic domain includes an ATP binding site and a threonine residue Thr308 (AKT1-Thr308, AKT2-Thr309, AKT3-Thr305), which is the necessary phosphorylation site for AKT activation. The C-terminal regulatory domain consists of 40 amino acids, holding a hydrophobic region, which containing the second phosphorylation site needed to activate AKT, namely the serine residue Ser473 (AKT1-Ser473, AKT2-Ser474, AKT3-Ser472). Although the three AKT subtypes are highly homologous, each exerts unique physiological functions. AKT1 is broadly distributed in all tissues of the body, mainly involved in cell growth, proliferation, angiogenesis and tumor cell invasiveness (Petrik et al., 2016). Found in Mammalian skeletal muscle, and adipose tissue, AKT2 are confirmed participate in cell growth and proliferation, and mediate glucose homeostasis (Garofalo et al., 2003). Relatively little is known about AKT3, it is crucial for brain development and the survival of malignant glioma cells (Ghoneum and Said, 2019).

### Signal Regulation of PI3K/AKT Pathway

#### Activation of PI3K/AKT Pathway

The activation of PI3K/AKT pathway begins with the activation of PI3K by RTK. Numerous cytokines or growth factors, such as fibroblast growth factor (FGF) and platelet-derived growth factor (PDGF), bind to the plasma membrane receptor RTK in response to stimulation by extracellular ligands, inducing receptor dimerization and cross-phosphorylation of tyrosine residues in intracellular domains. The regulatory subunit P85 binds to the phosphorylated tyrosine residue on the activated receptor through its SH2 domain. The catalytic subunit p110 is then recruited to form a fully active PI3K enzyme. Also, P110 subunit could be recruited independently of p85, like Ras-GTP, even other cohesive molecules as insulin receptor substrate (IRS). Besides, activation of Gα subunit could activate Src-dependent integrin signal transduction in PI3K. By unifying with the signal protein AKT and phosphatidylinositol dependent protein kinase 1 (PDK1) containing PH domain, Phosphatidylinositol (3,4,5)-trisphosphate (PIP3), the second messenger phosphorylated from Phosphatidylinositol (4,5)-disphosphate (PIP2) by P110, recruits inactive AKT and PDK1 from the cytoplasm to the cell membrane, thus enabling PDK1 to obtain catalytic activity. At the same time, the conformational change of AKT structure exposes the phosphorylation sites of Thr308 and Ser473, resulting the phosphorylation of Thr308 residues by PDK1 (Altomare et al., 2005; Wei et al., 2019). And the second phosphorylation at Ser473 at the carboxyl terminal is essential and mainly carried out by mechanistic target of rapamycin complex 2 (mTORC2) (Zeng et al., 2007). Many other kinases are known to phosphorylate AKT at Ser473, including PDK-1, integrin-linked kinase (ILK) or ILK-related kinases, and AKT itself (Memmott and Dennis, 2009). Binding proteins such as actin, extracellular signal-regulated kinase (ERK)1/2, heat shock protein (Hsp) 90 or Hsp27 are able to regulate the activity of AKT (Shanu and Komal, 2015). In addition, members of the family of PI3K-associated kinases, including DNA-dependent protein kinases (DNA-PK), can also phosphorylate AKT in Ser473 (Bellacosa et al., 2005).

As a downstream member of AKT, mTOR in turn acts as an activator for AKT activation. mTOR is linked to a regulatory-associated protein of mTOR (Raptor), and mammalian lethal with SEC13 protein 8 (mLST8) to form two multiprotein complexes with different functions, namely mTORC1 and mLST8 (Soliman, 2013). mTORC2 directly phosphorylates the hydrophobic motif Ser473 of AKT, and therefore enhances the activity of AKT kinase and promotes the phosphorylation of Thr308 by PDK1 (Laplante and Sabatini, 2012).

#### Negative Regulation of PI3K/AKT Pathway

Tumor suppressor, phosphatase and tensin homolog (PTEN) is a specific phosphatase with double activity (Satoru et al., 2018). Inactivation of AKT is mediated by the lipid phosphatase PTEN through dephosphorylation of PI(3,4,5)P3 to PI(4,5)P2, and by Src homology domain-containing inositol 5′-phosphatase 1 (SHIP1) due to conversion of PIP3 to PIP2. Negative regulation also be done by PH domain and leucine rich repeat protein phosphatase (PHLPP) and protein phosphatase 2 A (PP2A) which in turn dephosphorylate AKT at Ser473 and Thr308 respectively (Bertacchini, 2015).

#### Downstream Regulation of PI3K/AKT Pathway

After phosphorylated by the upstream signal, PI3K/AKT executes diverse biological actions by phosphorylating or forming complexes for a range of downstream molecules, such as the FoxO family members, GSK-3β, mTOR, and actin-related protein, among others (Mirdamadi et al., 2017; Matsuo et al., 2018). The activation of this signaling pathway and its downstream regulation are basically illustrated in Supplementary Figures S1 or S2. mTOR is the main downstream target of PI3K/AKT signal transduction and a key regulatory factor of cellular metabolism. AKT affects cell cycle progression by phosphorylating and inhibiting cyclin-dependent kinase inhibitors p21 and p27. And it modulates apoptosis through inhibiting Bcl2-antagonist of cell death (Bad), bcl-2-like protein 11 (BIM), caspase-9 and forkhead box protein O1 (FoxO1). Nuclear factor erythrocyte two related factor (Nrf2) is the main regulator of oxidative stress, which can promote the transcription of detoxification enzyme and antioxidant enzyme protein genes. Some drugs have been proved to activate PI3K/AKT/Nrf2 signaling, thereby reducing cognitive impairment and neurological dysfunction (Liang et al., 2018). In simple terms, PI3K/AKT pathway has been implicated in cell proliferation, glucose metabolism, cell survival, cell cycle, protein synthesis, and participates in neuronal morphology and plasticity through adjusting several downstream molecules. Imbalanced expression in solid tumors, immune-mediated diseases, cardiovascular diseases, diabetes, nervous system diseases and other diseases, PI3K/AKT pathway arouses the interest of many scholars act as a meaningful therapeutic target, worthy of a further exploration.

## Roles of PI3K/AKT Pathway in Alzheimer’s Disease

AD is a neurodegenerative disease positively concerned with age and associated with memory, cognitive impairment and behavioral changes (Martins et al., 2019). Two chiefly pathological features as following: senile plaque (SP) with amyloid protein (Aβ) as the core and abnormal high phosphorylation of Tau protein in brain nerve cells to form neurofibrillary tangles (NFT). With the aging of the population, the incidence of AD is increasing year by year, which brings a heavy burden to the society and families. Distinctly, the major problem facing researchers is to prevent and treat AD availably.

Although several hypotheses have been proposed after decades of research, the specific pathogenesis of AD is still obscure. The most common amyloid deposition hypothesis proposes that the self-assembly of misfolded amyloid peptides affects the structure and function of neurons, stimulates apoptosis, leading to synaptic dysfunction and neurodegeneration. It has been found that PI3K/AKT signal pathway is involved in the formation of two special pathological structures in AD (Do et al., 2014), so that activating the PI3K/AKT pathway may conduce to delay the progression of AD (Supplementary Figure S1). The activation of P3IK/AKT signal pathway is capable of protecting neurons against Aβ-induced neurotoxicity. Various targets downstream of this pathway are closely related to the occurrence and development of the disease. For example, the increase of GSK-3β activity is directly related to the increase of Aβ production and deposition, hyperphosphorylation of tau and the formation of NFT. During AKT phosphorylation, the phosphorylated protein of GSK-3β is inactivated at the Ser9 site, and therefore weakens the hyperphosphorylation of Tau protein, and inhibits the formation of NFT (Kitagishi et al., 2014).

Different from normal people, there is an evidently decrease in the number of neurons in some brain regions of AD patients. These changes were mainly caused by apoptosis induced by oxidative stress response and the excitatory toxicity of glutamate, and eventually lead to the occurrence of nervous system diseases. Studies have shown that cell death in AD is related to the changes in the expression of anti-apoptotic proteins (Bcl-2, Bcl-xL), which play an anti-apoptotic role by stabilizing the permeability of mitochondrial membrane and preventing the release of mitochondrial cytochrome C. Interestingly, the Pl3K/AKT signaling pathway can regulate the expression of mitochondrial membrane permeability protein Bcl-2, Bax and other proteins (Zeng et al., 2011). Other studies have shown that the repression of PI3K/AKT induces neuronal apoptosis through the mediation of P38 activation. In addition, overexpression of PTEN associated with the apoptosis, and the survival or death of neuronal cells may be partly attributed to the variations in PTEN expression (Satoru et al., 2018). Accordingly, the down-regulation of PTEN to promote the activation of AKT might be of great significance in maintaining its neuroprotective effects.

What else, synaptic plasticity of neurons could be adjusted by the PI3K/AKT pathway. In the animal model of AD, inhibition of PTEN is beneficial for preserving normal synaptic function and thereby improving cognition (Cui et al., 2017b). Some studies have confirmed that mTOR protein pathway in PI3K/AKT signaling is also involved in the development of neuronal dendrites and the formation of dendritic spines. The increase in synaptic plasticity is charac terized by long term potentiation (LTP) (Shane et al., 2019), which requires the activation of N-methyl-d-aspartic acid (NMDA) receptors, thereby promoting the insertion of *α*-amino-3-hydroxy-5-methyl-4-isoxazolpropionic acid (AMPA) receptors into the postsynaptic membrane. On the one hand, mTOR increases the expression of LTP-related proteins, and PI3K also binds to AMPA receptors and guides its distribution on the membrane (Parkinson and Hanley, 2018). On the other hand, NMDA receptor activation also promotes PI3K activation. In addition, AKT immobilizes phosphatidylinositol (PI) to the postsynaptic membrane to recruit the docking protein of AMPA receptor and promote the fixation of AMPA receptor in the postsynaptic membrane (Spinelli et al., 2019). As a result, PI3K/AKT/mTOR signaling pathway regulates neuronal synaptic plasticity at multiple nodes.

## Roles of PI3K/AKT Pathway in Parkinson’s Disease

PD is the second largest neurodegenerative disease after AD, also known as idiopathic tremor paralysis. It is characterized by the degeneration and deletion of dopaminergic neurons in substantia nigra (SN) and the formation of eosinophilic inclusion body (Lewy bodies, LBs), as well as the production of neuroinflammation. For decades, though, there has been a great deal of research on PD, drugs on effectively inhibiting or reversing the development of PD are still an assumption except the treatment of temporary improvement of symptoms, so the treatment of PD is more difficult than it seems. As for the pathogenesis of PD, scientists have successively proposed hypotheses including oxidative stress mitochondrial damage, excitatory amino acid toxicity, inflammatory response, and abnormal deposition of *α* synuclein. However, most of the pathogenic factors have not been confirmed. At present, the abnormal aggregation of fibrillation and *α*-synuclein is considered to be the key factor for the cascade of pathological events in PD. LBs is mainly composed of misfolded proteins, such as synuclein, tubulin, and amyloid precursor protein. Mitogen-activated protein kinases (MAPKs) like AKT/ERK, found in cells, participate in the removal of these proteins through phosphorylation and dephosphorylation. Intracellular deposition of alpha synuclein leads to neuronal degeneration and apoptosis. Autophagy degrade synuclein and resist the deposition of synuclein in Lewy, so it is usually increased in PD patients (Chen et al., 2017).

The loss of dopaminergic neurons in the SN and striatum caused by apoptosis is an important cause of PD. Scholars manifested that AKT and phosphorylated AKT are significantly reduced in the substantia nigra compacta (SNpc) of PD patients (Luo et al., 2019). GSK-3 is widely expressed in the central nervous system, but abnormally in PD (Zhang et al., 2016b). Caspase-3 plays a key role in apoptosis and as a key effector in all apoptosis pathways. During the pathogenesis of PD, GSK-3 activation up-regulates the content of caspase-3 in the dopaminergic nerve, leading the apoptosis of dopaminergic neurons. Experiments show that AKT could inhibit the activity of GSK-3 by phosphorylating Ser21 of GSK-3α or Ser9 of GSK-3β (Liying et al., 2018). As a downstream pathway of AKT, IKK/IκBα/NF-κB pathway is one of the vital pathways for cell survival (Yan et al., 2019). Consequently, the activation of PI3K/AKT pathway facilitates the survival and growth of dopamine neurons by inhibiting apoptosis. Besides, knockout of PTEN make a contribution to neuroprotection and promotion of the rapid growth of dopaminergic (DA) neurons (Wang et al., 2017).

Oxidative stress, inducing neuronal cell death and apoptosis through intracellular calcium overload lipid peroxidation DNA damage and excitatory toxicity, also contributes to the onset of PD. The PI3K/AKT pathway influences oxidative stress by modulating downstream molecular targets such as GSK-3, mTOR and FoxO3a. Decreasing the mTOR activity may lead to neurodegeneration. The stress response protein regulated in development and DNA damage responses 1 (REDD1) is up-regulated in dopaminergic neurons in PD patients and can modulate the activity of mTOR (Chong et al., 2012). When Parkin (a mutation-prone gene associated with PD) is abnormally expressed, the PI3K/AKT/FoxO3a pathway is blocked, resulting in the imbalance of oxidative stress and ultimately the occurrence of PD (Gong et al., 2018). Additionally the relationship between the above mentioned PI3K/AKT signaling pathway and PD is clearly sorted out in Supplementary Figure S2.

## Natural Products for Prevention and Treatment of Alzheimer’s Disease Based on PI3K/AKT Pathawy

**FIGURE 1:**
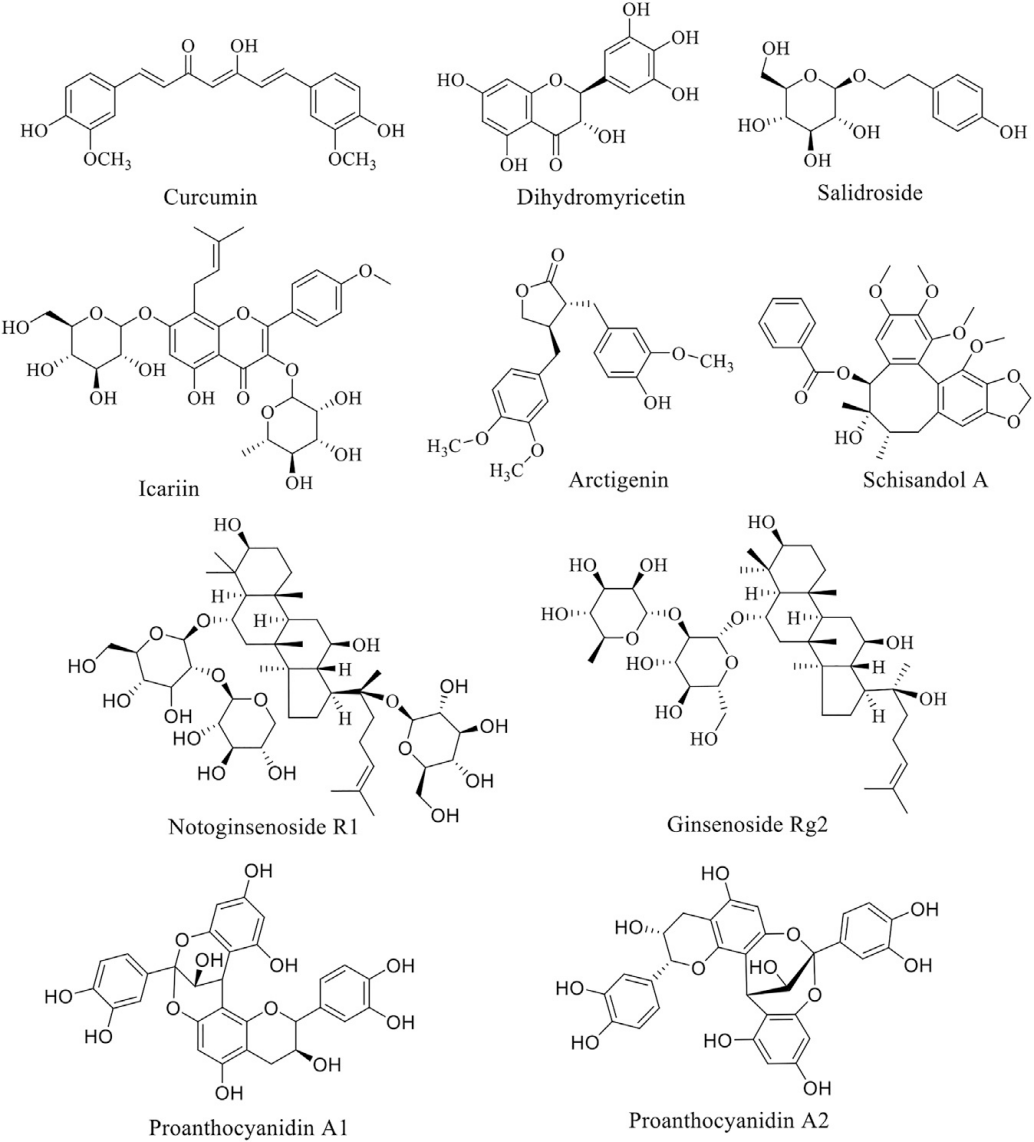
Structure of anti-PD natural compounds.

Natural products have attracted the attention of researchers by virtue of their natural advantages such as wide variety, wide source and wide function spectrum. Structurally classified into flavonoids, alkaloids, phenylpropanoids, glycosides, and others, natural products are widely distributed in nature and commonly found in herbs, fruits and vegetables. Interestingly, almost every natural product has a variety of pharmacological properties, covering anti-tumor, antioxidant, antibacterial, anti-inflammatory, anti-diabetic, hypoglycemic, neuroprotective, etc. Additionally, natural products are generally less toxic and very safe, hence becoming an important alternative source of many types of drugs. Over the years, AD has become the fourth leading cause of death after cardiovascular disease, cancer and stroke. Under the circumstance of poor progress in synthetic drug research, natural product anti-AD therapy has become an attractive direction of exploration. Natural products such as curcumin, dihydromyricetin and salidroside have been found to have eminently preventive and therapeutic effects on AD through PI3K/AKT pathway. These natural products are summarized below, and the structure of natural products is listed (Figure 1). The experimental cell, animals, dose and time used in natural product research were summarized in Table 1, providing reference for readers.

**TABLE 1:** Summary of some natural products with AD and PD therapeutic potential.

Natural products	Diseases	*In-vitro*/vivo models/human trial	Dose and period of treatment	Treatment time	Mechanism	Activation or inhibition of PI3K/AKT pathway	References
Curcumin	AD	N2a/WT cells	5 μM for cell line and 1or 0.16 g/kg for animals	6 months	Decrease in Caveolin-1, inactivation of GSK-3 and inhibition of abnormal excessive tau phosphorylation, bax and increase in Bcl-2	Activation	Sun et al. (2017)
	PD	APP/PS1 transgenic mice					
Dihydromyricetin	AD	D-gal-induced aging rat model	The doses of 100 and 200 mg/kg	5 weeks	Decrease the expression of caspase-3, p53 and p62, up-regulate Bcl-2 and SIRT1 activity, suppress aging-related astrocyte activation and inhibiting mTOR signal pathway as well as down-regulate miR-34a	Activation	Kou et al. (2016)
	PD	Sprague-dawley rats					
Salidroside	AD	Transgenic drosophila AD models	Concentration of 50,100,200 μM	24 h	Decrease aβ levels and aβ deposition, increase AKT phosphorylation level *p*-mTOR or p-p70S6K level	Activation	Zhang et al., (2016a)
		C57BL mice	Concentration of 0.3 mg/ml	2–12 months	Reduce the aggregation of aβ and improve synaptic structure. Activation of PI3K/AKT/mTOR signaling pathway		Wang et al. (2020)
		APP/PS1 transgenic mice					
Arctigenin	AD	ICR mice	The doses of 10, 40, or 150 mg/kg for animals	16 days	Inhibit the production of phosphorylated tau, and inhibition of the PI3K/akt/gsk-3β signaling pathway	Activation	Qi et al. (2017)
Schizandrol A	AD	SH-SY5Y cells or primary hippocampal neurons	Concentration of 2 μg/ml	24 h	Suppress the ratio of Bax/Bcl-2 and caspase-3, increase of p62, and decrease of LC3-II/LC3-I, Beclin-1, decreased ratios of p-Tau/Tau	Activation	Song et al. (2020)
Ginsenoside Rg2	AD	Male sprague-dawley rats	The doses of 25,50,100 mg/kg/day	6 days	Increase the Bcl-2/Bax ratio, attenuate the cleavage of caspase-3, and enhance the phosphorylation of AKT.	Activation	Cui et al., (2020)
Notoginsenoside R1	AD	C57BL/6J mice and APP/PS1 mice	Dose of 5 mg/kg/day	6 months	Improve cell viability, reduce the cleavage of Navb2 by BACE1 suppression, and also correct the abnormal distribution of Nav1.1a	Activation	Hu et al. (2020)
Fructus broussonetiae	AD	APP/PS1 double-transgenic mice	0.1, 0.15,0.3 g/ml for mice, and 150 mg/kg for rabbits	2 months	Increase *p*-AKT and *ß*-catenin signaling, decrease caspase-3,caspase-9, and levels of aβ and phosphorylate tau proteins	Activation	Li Y.-h. et al. (2020)
		Rabbits		10 days			
Icariin	AD	SAMP8 mice model of AD	The doses of 60 mg/kg	22 days	Down-regulate the expression of BACE1 to reduce the expression of cytotoxic A*β*_1-42_, and increase the Bcl-2/Bax ratio	Activation	Wu et al. (2020)
	PD	Ovariectomized PD mice, doparminergic MES23.5 cells	The doses of 50/100/200 mg/kg	13 days	Increase the DA content, the Bcl-2 and attenuate the increase of bax and caspase-3 protein levels, active PI3K/AKT or MEK/ERK signaling pathway		Chen et al. (2017)
Baicalein	AD	PC12 cells	Concentration of 40/60/80 μM	2 days	Increase the cell viability and niacin level. Inhibit A*β*_25–35_-induce cytotoxicity by restraining the levels of ROS, and increasing the level of MMP and mitochondrial respiratory complex I	Activation	Gao et al. (2020)
	PD	Sprague-dawley rats	The doses of 30 mg/kg/day	7 days	Reduce procaspase-1, caspase-3 and elevate active caspase-1 levels		Zhao et al. (2017)
				9 days			
Baicalin	PD	Sprague-dawley rats	The doses of 25 mg/kg	4 weeks	Increase the expression of mTOR, *p*-mTOR, AKT, *p*-AKT, GSK-3β, and *p*-GSK-3β	Activation	Zhai et al. (2019)
Apigenin	PD	SH-SY5Y cells, C57BL/6 mice	10,20,40,100 μM for cells and 50 mg/kg/day for mice	2h and 15 days	Upregulate the p-PI3K/PI3K ratio and *p*-AKT/AKT ratio while downregulate Bax/Bcl-2 ratio and caspase-3 activity, improve the lesioned neurobehavior	Activation	Hu et al. (2018)
Amentoflavone	PD	C57BL/6 mice	The doses of 50 mg/kg/day	15 days	Elevate the viability and alleviate apoptosis, restore the decreased TH expression, inhibite the activation of caspase-3 and p21 but increase the Bcl-2/Bax ratio, activate PI3K/AKT and ERK signaling pathways	Activation	Cao et al. (2017)
Puerarin	PD	SH-SY5Y cells, sprague–Dawley rats	10,50,100,150,200 µM for cells and 50 mg/kg for animal	12 h, 7 days	Elevate the viability, mitigate intracellular oxidative stress and ROS, up-regulate TH and VMAT2 expressions, and dopamine levels, alleviate behavioral defects of PD.	Activation	Zhang et al. (2014)
Tovophyllin A	PD	Primary cortical neurons, C57BL/6 mice	The doses of 40 mg/kg	24 h	Alleviate MPTP-induced behavioral dysfunctions and DA neuron loss, increase the phosphorylation of AKT and GSK-3β	Activation	Huang et al. (2020)
Berberine	PD	SH-SY5Y neuroblastoma cells	Concentration of 10^–5^,10^–4^,10^–3^, 10^–2^,10^–1^,1,10, and 100 µM	24 h	Up-regulate the Bcl-2, downregulate the bax and caspase-3, activate PI3K/AKT signaling pathway	Activation	Deng et al. (2020)
Schisantherin	AD	Differentiated rat pheochromocytoma PC12 cells	SCH (50 μM) and NKT (10 μM)	24 h	Activate the PI3K/AKT/GSK-3β/mTOR pathway, and inflammatory related proteins such as NF-κB, IKK, IL-1β, IL-6 and TNF-α are decreased	Activation	Qi et al. (2020)
Schisandrol A	PD	C57BL/6J mice	The doses of 10,20,30 mg/kg	2 weeks	Decrease the focal encephalomalacia and inhibite the striatal degeneration, enhance the PI3K/AKT pathway, inhibit the IKK/IκBα/NF-κB pathway, reduce neuronal inflammation and oxidative stress, and enhance the survival of DA neurons in the brain of mice	Activation	Yan et al. (2019)
Paeoniflorin	PD	C57BL/6 mice	The doses of 7.5,15,30 mg/kg	1 week	Prevent the TH and DAT protein decrease induced by MPTP, prevent the striatal *p*-AKT (Ser473) protein decrease, attenuate caspase-3 and caspase-9 activation	Activation	Zhao et al. (2017)
Cannabidiol	PD	The human neuroblastoma cell line SH-SY5Y	Concentration of 10 μM	24 or 48 h	Decrease LC3-II levels, activate the ERK and AKT/mTOR pathways and modulate autophagy	Activation	Gugliandolo et al. (2020)
Lycium barbarum polysaccharide	PD	C57BL/6 mice	The doses of 100 or 200 mg/kg	21 days	Up-regulate the TH level, inhibit the oxidative stress in the midbrain, and inhibit the aggregation of *a*-synuclein, downregulated LC3-II and beclin expression, activate the AKT/mTOR pathway through inhibiting PTEN.	Activation	Wang et al. (2018b)
*Astragalus* polysaccharides	PD	PC12 cell	Concentration of 50,100,200 μM	24 h	Promote the phosphorylation of AKT and mTOR, and up-regulate the expression of PTEN.	Activation	Tan et al. (2020)
Crocin	PD	Adult male wistar rats	The doses of 30 mg/ml/day	1 month	Increase active form of AKT, reduce expression and activity of FoxO3 and GSK-3β, elevate miRNA-221 expression, decrease pro-apoptotic caspase-9 and enhance anti-apoptotic Bcl-2	Activation	Salama et al. (2020)
Asiatic acid	PD	C57BL/6 mice	The doses of 25,50,100 mg/kg		Increase the phosphorylation of PI3K, AKT, GSK-3β and mTOR, inhibition of JNK, ERK and P38 MAPK-mediated signaling pathways	Activation	Nataraj et al. (2017)

Aβ, amyloid-β; AKT, protein kinase B; BACE1, amyloid precursor protein cleaving enzyme one; Bad, Bcl-xL/Bcl-2-associated death promoter homologue; caspase-3, cysteine protease protein; ERK, extracellular signal-regulated kinase; GSK-3β, glycogen synthase kinase-3β; HO-1, heme oxygenase-1; IKK, IκB kinase; IL-1β, interleukin-1β; IL-6, interleukin-6; JNK, c-Jun NH(2)-terminal kinase; LC3-II/LC3-I, the autophagosome-associated protein; MAPK, mitogen-activated protein kinases; MMP, mitochondrial membrane potential; MPTP, 1-methyl-4-phenyl-1,2,3,6-tetrahydropyridine; mTOR, mammalian target of rapamycin; NF-κB, nuclear factor-kappa B; p53, tumor suppressor gene; p62, the atypical protein kinase C-interacting protein; PI3K, phosphoinositide 3-kinase; PTEN, phosphatase and tensin homolog; SD, Sprague-Dawley; SIRT1, Silent mating type information regulation two homolog-1; TNF-α, tumor necrosis factor-alpha.

### Flavonoids

Curcumin is a rare diketone compound in the plant kingdom, mainly extracted from the rhizomes of some plants in Zingiberaceae and Araceae (Mario et al., 2016). The protective property of curcumin on neuronal degeneration caused by mitochondrial dysfunction and the resulting oxidative stress, inflammation and apoptosis of nerve cells has been demonstrated. And the neuroprotection pathogenesis of curcumin is mediated by two momentous signaling pathways, PI3K/AKT/GSK-3 or PI3K/AKT/cAMP response element-binding protein (CREB)/brain-derived neurotrophic factor (BDNF) (Kandezi et al., 2020). By regulating above pathway, curcumin inhibits the glutamate-induced release of mitochondrial cytochrome C, the activation of caspse-3, a key enzyme for cell apoptosis, as well as reduces pro-inflammatory biomarkers (interferon-α (IFN-α), tumor necrosis factor-α (TNF-α), interleukin (IL)-8) and increases anti-inflammatory cytokines/compounds (IL-10, IL-1) (Wolkmer et al., 2013). It is noteworthy that the increasing clinical data indicate that curcumin is expected to be a standard candidate for neuroprotective agent (Voulgaropoulou et al., 2019). Yet not all clinical trials are positive. Some clinical studies show that curcumin has a beneficial effect on cognitive improvement of AD (Cox et al., 2015; Small et al., 2018). Whereas another suggest that curcumin has no cognitive-enhancing properties (Ringman et al., 2012). Curcumin reduces Aβ deposition in the brain, which is supported by neuroimaging (Small et al., 2018). While in another clinical trial, the results are misty (Ringman et al., 2012).

Dihydromyricetin (DHM), also called ampelopsin, is a dihydroflavonol compound, has been found to markedly rescued apoptosis of neurons in hippocampus of D-gal-induced brain aging model of rats, improving learning and memory impairment (Kou et al., 2016). It activates hemeoxygenase-1 (HO-1) in pheochromocytoma 12 (PC 12) cells, and increases the expression of HO-1 or enzyme activity, thus againsts H_2_O_2_ and 6-OHDA-induced neurotoxicity in PC12 cells. The protection of ampelopsin is link to inhibition of rwactive oxygen species (ROS) formation, expression of poly ADP-ribose polymerase (PARP) and caspase-3, inhibition of p38, MAPK phosphorylation and up-regulation of HO-1 expression. Interestingly, the increase in HO-1 expression is mainly due to increased phosphorylation levels of ERK and AKT (Kou et al., 2012). Based on AKT and ERK1/2 signaling, DHM also dose-dependently attenuated sodium nitroprusside induced PC12 cell damage (Liao et al., 2014). In addition, DHM up-regulates Bcl-2, down-regulates cleaved caspase3 and Bax, increases antioxidant capacity and inhibits apoptosis (Mu et al., 2016). In another study, DMY improves glucose metabolism of PC12 cells stimulated by endogenous toxic compound methylglyoxal through AMPK/glucose transporter 4 (GLUT4) signaling pathway, inhibiting oxidative stress, and protecting mitochondria of cells (Jiang et al., 2014a). Moreover, DHM has been confirmed to execute its protective function through GSK-3β/Nrf2/antioxidant response element (ARE) signal pathways (Kou et al., 2015).

Icariin (ICA), a flavonol isolated from *Epimedium perralderianum* Coss (family *Berberidacea*, genus *Epimedium*), is the main active component of *Epimedium perralderianum*. It had previously been observed that ICA alleviated symptoms in AD model animals, which may be caused by increased expression of insulin-like growth factor (IGF) and BDNF, thereby activating PI3K/AKT pathway, inhibiting production of Aβ and tau protein phosphorylation (Wu et al., 2012). A recent *in vivo* study suggested that the promotion effect of ICA on proliferation and differentiation of hippocampal neural stem cells in AD model is related to the BDNF-tyrosine kinase B (TrkB)-ERK/AKT signaling pathway (Lu et al., 2020). Moreover, ICA treatment significantly enhanced proteasome-dependent degradation of PTEN and protected SK-N-MC cells from Aβ-induced insulin resistance (Zou et al., 2020). In addition, by down-regulating the expression of β-site amyloid precursor protein (APP) cleavage enzyme 1, icariin could decrease the deposition of β-amyloid peptide (Wu et al., 2020).

### Phenylpropanoids

Phenylpropanoid compounds incorporate simple phenylpropanoid, coumarin and lignans. Salidroside (Sal), belonging to phenylpropanoid glycoside, is a bioactive substance mainly extracted from traditional herbs such as *Rhodiola coccinea* (Royle) Boriss., be of a species in the genus *Rhodiola* (family Crassulaceae). In recent years, Sal has been reported to exhibit great neuroprotection, including antioxidant, anti-inflammatory, anti-apoptosis and regulation of multiple signal pathways and key molecules (Fan et al., 2020). Zhang et al., in their study on the therapeutic potential of Sal for AD, pointed out that Sal alleviate pathological progression in AD models by reducing amyloidosis and activating PI3K/AKT signaling, suggesting the potential role of Sal in the prevention and treatment of AD due to its neuroprotective property. In addition, mTOR has been found to be a mammalian target that plays an important role in the formation of AD related memories (Zhang et al., 2016a). In a subsequently study, results are similar to Bei et al. However, the alteration of mTOR might be influenced by different animal models or drug delivery methods, and the exact mechanisms remain to be investigated (Wang et al., 2020). Additionally, Sal reduces inflammation and brain injury after middle cerebral artery occlusion in rats via PI3K/PKB/Nrf2/NFκB signaling pathway (Zhang et al., 2019a). In an experiment to test the cognitive effect of Sal on AD mice, the researchers demonstrated that Sal reduced the expression of TNF-α and IL-6 cytokine inflammatory factors, improving cognitive function in AD mice. The authors suggested that this protective effect may be related to changes in the level of free radicals in the hippocam pus (Li et al., 2018). But it only limited to the guess rather than further inquiry. Structurally, Sal is linked by a glycosidic group to an aglycon (tyrosol) via a *β*-glycosidic bond. The polyhydroxyl structure of Sal gives it a strong polarity, thereby limiting its diffusion and absorption through the body (Cheng et al., 2012). In order to overcome the above problems, Yang et al. synthesized twenty-six novel derivatives of Sal and evaluated their cell protective effects in CoCl_2_-treated PC12 cells (Yang et al., 2021). Among the twenty-six salidroside derivatives, five of them showed stronger cell protective effect than Sal (EC_50_, 0.30 µM) under the same condition. Among them, pOBz had a more significant protective effect (EC_50_, 0.038 µM). Although the mechanism studies found that pOBz reduced monoamine oxidase (MAO) activity and played a neuroprotective role after MCAO reperfusion in rats, and also inhibited the expression of C3 protein in the brain after cerebral ischemia reperfusion injury, these studies were obviously insufficient. Whether the action mechanism of pOBz and other derivatives is the same as Sal remain to be further studied, and their molecular targets also need to be further studied.

Arctigenin is a phenylpropanoid dibenzylbutyrolactone lignan compound, naturally found in *Arctium lappa L.* (family Compositae, genus *Arctium*). Qi et al. firstly elucidated that arctigenin (10, 40, or 150 mg/kg, orally) attenuates the level of phosphorylated tau protein expression in the hippocampus through PI3K/AKT/GSK-3β signaling pathway in Aβ-induced AD mice, hence successfully providing protection against learning and memory deficits (Qi et al., 2017). However, different routes of administration affect the bioavailability of arctigenin *in vivo*. Pharmacokinetic investigations have shown that the oral administration of arctigenin will cause large amounts of primary metabolism, which would affect its *in vivo* and clinical efficacy. So it is suggested that Arctigenin should be administered through the intranasal route for the treatment of central nervous system dysfunction (Gao et al., 2018). This suggests that monitoring pharmacokinetics is crucial for better understanding the role of arctigenin. Due to the few researches, clinical trials of arctigenin are rather limited. Therefore the anti-AD effect of arctigenin has not been clinically confirmed. In brief, sufficient research data is required to justify the need for clinical trials. Schizandrol A (SchA), a type of lignans, is a natural active ingredient extracted from *Schisandra chinensis* (Turcz.) Baill. (family Schisandraceae, genus *Schisandra*), the Chinese herb fruit. In a study of the neuroprotective effects of SchA in AD cell models, researchers demonstrated that SchA swimmingly reduces the decrease of living cells, the increase of the number of apoptotic cells, the expresstion of pro-apoptotic proteins and the changes of oxidative stress markers induced by A*β*_1-42_. Moreover, SchA inhibited the increase of microtubule-associated protein 1A/1B-light chain 3 (LC3)-II/LC3-I and Beclin-1 and the decrease of p62. It revealed that SchA perhaps to be a new drug for the prevention and treatment of AD (Song et al., 2020). SchA has been shown to inhibit ischemia-induced neuronal autophagy via AMPK/mTOR pathway (Wang et al., 2019). A comparative experiment was carried out that SchA (50 μM) combined with norepinephrine (NE, 10 μM) work better than alone (Qi et al., 2020).

### Saponins

Ginsenosides are the main bioactive components of *Panax ginseng* C.A.Mey. (family Araliaceae, genus *Panax*) with the functions of eliminating free radicals, anti-oxidation, delaying cell aging, protecting nervous system, and improving memory of the elderly, becoming the hot candidate drugs for the treatment of central nervous system diseases. Investigators have showed that ginsenosides enhance the expression of synaptic-related proteins and synaptic plasticity, up-regulate the expression of CREB and BDNF, reduce apoptosis and AD pathology-like protein expression, scavenge free radicals and protect nerve cells (Lulin et al., 2017). A study on HT22 cells and APPSW SH-SY5Y cells demonstrated that RG1 promoted *a*-secretase cleavage of APP by activating ERK/MAPK and PI3K/AKT pathway (Shi et al., 2012). Li et al. also illustrated via experiments that ginsenosides improve memory and reduce the content of A*β*_1-42_ and p-tau in AD mice, and the anti-AD effect of ginsenoprotein is mediated by activating PI3K/AKT signal pathway (Li et al., 2016). Other studies have confirmed that ginsenoside Rg2, one of the most important active components of ginsenosides, significantly inhibits the apoptosis of Aβ treated PC12 cells by up-regulating the PI3K/AKT signaling pathway, which may be caused by the decrease of ROS and intracellular Ca^2+^ concentration by ginsenoside, the activation of autophagy pathway, the up-regulation of Bcl-2 and the down-regulation of Bax (Cui et al., 2017a). Taken together, ginsenosides are deemed as potential new drugs for the treatment of AD (Cui et al., 2020).

Notoginsenoside R1 also possesses a certain repair effect on AD (Hu et al., 2020). Notoginseng R1 as reported conspicuously improved the cell damage induced via A*β*_25–35_ by increasing cell activity, inhibiting oxidative stress and the activation of MAPK signaling pathway (Ma, 2014). Meng et al. have found that the neuroprotection of notoginsenoside R1 is related to estrogen receptor dependence, and the up-regulation of antioxidant enzymes is associated to AKT and ERK1/2, as well as Nrf2 pathway (Meng et al., 2014).

### Non-flavonoid Polyphenols

Being a naturally occurring stilbene, piceatannol (PT) has been reported to possess antioxidant, anti-inflammatory, anti-diabetic and neuroprotective effects (Zhang et al., 2018). In an early study, the effects of PT and pterostilbene (PS) against Aβ-induced apoptosis in PC12 cells were evaluated. The results show that PT and PS have obvious anti-apoptotic activity. PT up-regulates the PI3K/AKT/BAD signaling pathway, further inhibits the expression of Bcl-2/Bax, the cleavage of caspase-9, caspase-3 and PARP. While PS promotes phosphorylation of AKT without affecting other factors (Fu et al., 2016). What’s more, the active fraction derived from *Litchi chinensis* Sonn. (family Sapindaceae, genus *Litchi*) seed can improve the cognitive function and behavior of AD model rats through decreasing Aβ fibril formation and Tau hyperphosphorylation. Catechin, proanthocyanidin A1 and proanthocyanidin A2 polyphenols isolated from seed of *Litchi chinensis* Sonn. inhibit hyperphosphorylated Tau protein by up-regulating IRS-1/PI3K/AKT and down-regulating GSK-3 (Fu et al., 2016).

### Others

Being commonly used spices, *Zanthoxylum bungeanum* Maxim. (Z. bungeanum) (family Rutaceae, genus *Zanthoxylum*) and its ingredients possess anti-inflammatory, antioxidant and neuroprotective effects (Deng et al., 2019). Zhao et al. demonstrated for the first time that *Zanthoxylum bungeanum* Maxim water extract (WEZ) and volatile oil extract (VOZ) inhibit apoptosis and oxidative stress by activating PI3K/AKT/Nrf2 signal pathway, attenuating impairment of d-galactose-induced aging mice. Unfortunately, the specific active ingredients have not been reported (Zhao et al., 2020). Evaluated the functions of *Broussonetia papyrifera (L.) L'Hér. ex Vent.* (family Moraceae, genus *Broussonetia*) in both mouse and cell models of AD, Li and colleagues found that Broussonetia papyrifera (L.) L'Hér. ex Vent. up-regulates AKT and *β*-catenin signaling pathways in the pretreated PC12 cells (Li Y.-h. et al., 2020). Same as other traditional Chinese herbal medicine with a variety of components, which component plays a critical role in AD is unclear.

## Natural Products for Prevention and Treatment of Parkinson’s Disease Based on PI3K/AKT Pathawy

Due to the complex pathogenesis of PD, there is no specific drug for it so far. The research and development of anti-PD drugs have attracted great attention from the medical community all over the world. In recent years, with the deepening of the research on neurophysiology, biochemical and pharmacology of the elderly, the research on the development of natural products against PD is in hot progress. Thanks to the efforts of many scholars, many natural products have been proved to have good effects on the treatment of PD. Natural flavonoids products, such as icariin, baicalein, other products like berberine, paeoniflorin, etc. have achieved great success in PD model animals and *in vitro* cell experiments.

### Flavonoids

**FIGURE 2:**
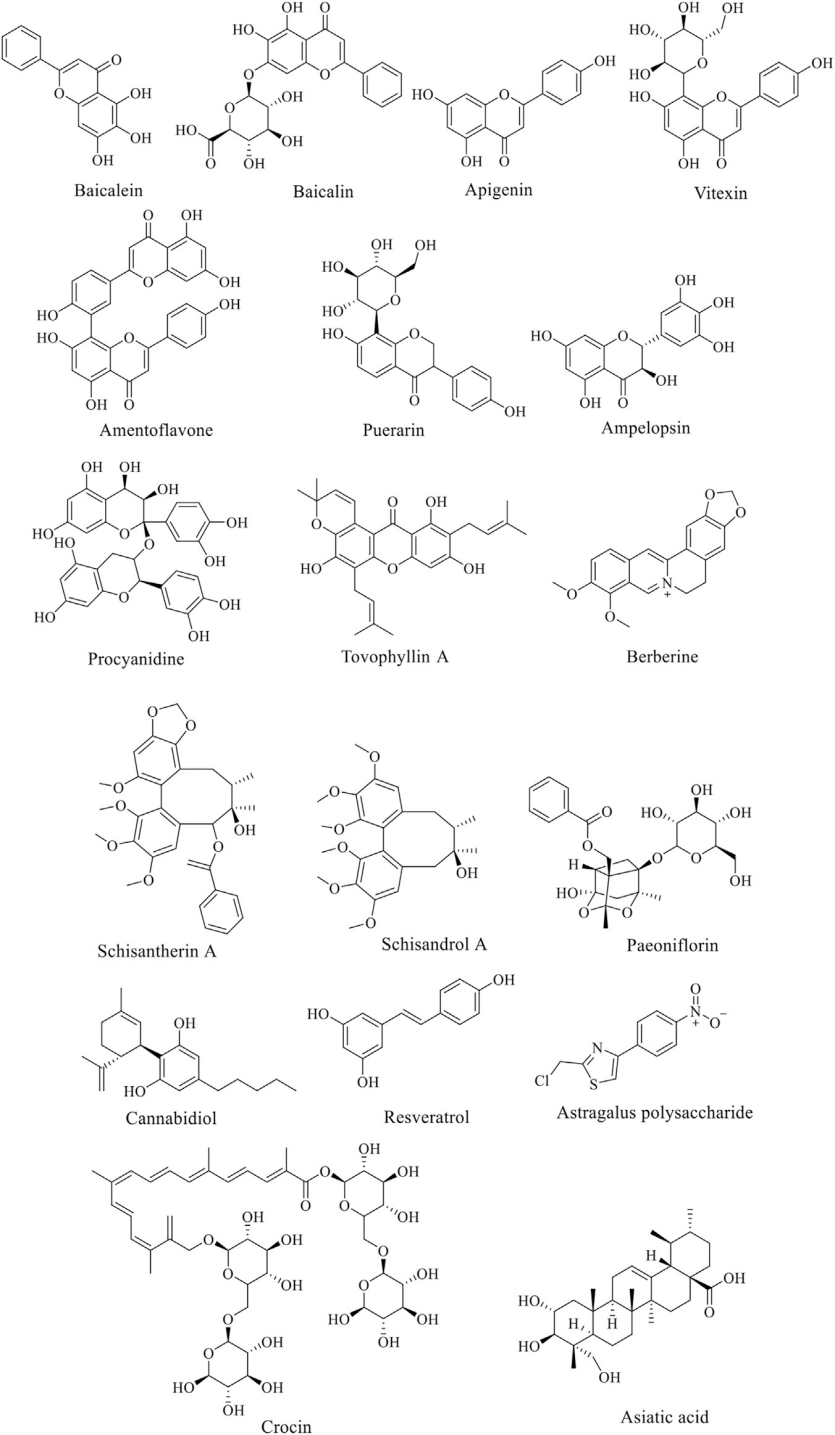
Structure of anti-AD natural compounds.

Baicalein is a bioactive flavonoid monomer compound isolated from *Scutellaria baicalensis* Georgi (family Lamiaceae, genus *Scutellaria*). Evidences suggest that baicalein have neuroprotective functions in addition to antioxidation such as alleviate the symptoms of AD and PD (Li et al., 2017). According to previous studies, baicalein was found to have a neuroprotective effect on acrolein-induced neurotoxicity at multiple levels (Zhao et al., 2017). And baicalein pretreatment significantly inhibited 6-hydroxydopamine (6-OHDA)-induced apoptosis (Zhu et al., 2019). Zhang et al. showed that the anti-PD function of baicalein is probably related to the activation of Nrf2/HO-1, protein kinase C (PKC) and PI3K/AKT signaling pathways (Zhang et al., 2012). Baicalin has one more glucuronide in the seven hydroxyl group than baicalein (Figure 2), which inhibit 6-OHDA-induced apoptosis of substantia nigra neurons in PD rats through mTOR/AKT/GSK-3β pathway (Zhai et al., 2019). Apigenin has been shown to remarkably reduce the effects of 1-methyl-4-phenylpyridine (MPP^+^)-induced apoptosis in experimental models of PD. Furthermore, the glucoside derivative Vitexin of apigenin and several other flavonoid species showed similar protective effects on PD cells and mouse models (Ming et al., 2018).

Amentoflavone (AF) is an effective component of *Selaginella tamariscina* (P.Beauv.) Spring (family Selaginellaceae, genus *Selaginella*). In an *in vitro* of PD model study by Cao et al., AF enhanced PI3K phosphorylation, and reduced the loss of cell viability induced by MPP^+^, without obvious cytotoxicity. It also inhibits the activation of caspase-3 and p21, but increases the ratio of Bcl-2/Bax. *In vivo*, AF significantly reduces the loss of dopaminergic neurons in SNpc and striatal fibers induced by 1-methyl-4-phenyl-1,2,3,6-tetrahydropyridine (MPTP), and enhances the activation of PI3K and AKT and the ratio of Bcl-2/Bax in SN (Cao et al., 2017). Similarly, the PD mouse experiment of icariin obtained a similar conclusion as AF (Chen et al., 2017). Puerarin (PUE) is a vital isoflavone compound in traditional Chinese medicine *Pueraria lobata*. Zhang et al. have shown that PUE could reduce the protection of dopaminergic neurons from rotenone toxicity in PD animal model of PD by activating PI3K/AKT signal pathway, and decrease the overexpression of abnormal proteins in PD animal model (Zhang et al., 2014).

According to the reports, Ampelopsin increases the antioxidant and anti-apoptotic activity of cells through ERK1/2 and AKT signaling pathways, thus protecting neurons (Coria et al., 2019; Guo et al., 2020). Likewise, Procyanidine (PC), widely found in *Vitis vinifera* L. (family Vitaceae, genus *Vitis*) and other plants, tellingly scavenge free radicals, inhibits neuronal apoptosis and protects mitochondria (Zhang et al., 2019b). In recent years, Tovophyllin A (TA) has been reported to play a useful role in the treatment of neurodegenerative diseases. Some studies have found that TA has significant neuroprotective effects on primary cortical neurons injured by MPP^+^/paraquat and PD mouse model induced by MPTP. The mechanism is confirmed to be related to the apoptosis signal pathway of AKT/GSK-3 cells, but it still need a further explored (Huang et al., 2020). It has also been reported that curcumin inhibits the activation of astrocytes and microglia (Fu et al., 2015), and protects mitochondrial membrane potential from oxidative damage to dopaminergic neurons (Ma and Guo, 2017). Curcumin could conspicuously improve the oxidative damage of dopaminergic neurons induced by oxidative stress in the compact part of substantia nigra of rats via activating AKT/Nrf2 signal pathway, demonstrating great therapeutic impact on the experimental model of PD (Cui et al., 2016).

### Alkaloids

Berberine (BBR), an alkaloid in the medicinal plants such as *coptis chinensis* Franch. (family Ranunculaceae, genus *Coptis*) and *Phellodendron amurense* Rupr. (family Rutaceae, genus *Phellodendron*), is proved to be of anti-oxidative stress and anti-apoptotic role in central nervous system diseases. The low concentration of BBR can significantly reduce apoptosis induced by Cytomegalovirus in cultured spiral ganglion cells via NMDAR1/Nox3, decreasing mitochondrial ROS generation (Zhuang et al., 2018). It have be reported that BBR inhibits ROS levels, mitochondrial dysfunction and mitochondrial autophagy by PI3K/AKT/mTOR signal pathway, thus protecting PC12 cells from oxidative damage (Zhang et al., 2017). In another research, a new concept emerged that BBR can attenuate the cytotoxicity induced by tert-butyl peroxide (t-BHP) (Deng et al., 2020). Han et al. demonstrated once again that berberine protects SH-SY5Y cells treated with rotenone through antioxidant and activation of the PI3K/AKT signaling pathway (Li Z. et al., 2020).

### Phenylpropanoids

Lignans are natural compounds polymerized from 2-molecular phenylpropanoid derivatives. Schisandra chinensis lignans (SCL) of include Schisandra Ester A, B, C and D. Previous studies have suggested that the protective effect of SCL on cerebral ischemic nerve injury may be achieved through PI3K/AKT pathway. Meso-dihydroguaiaretic acid, schiarisanrin A and heteroclitin D, lignans isolated from the root of *Kadsura coccinea* (Lem.) A.C.Sm. (family Schisandraceae, genus *Kadsura*), significantly inhibit LPS-induced neuronal injury through the same signal pathway (Jiang et al., 2014b). Schisantherin A has been reported to enhance antioxidant stress, inhibit the overproduction of nitric oxide and prevent the loss of dopaminergic neurons stimulated by 6-OHDA in zebrafish (Zhang et al., 2015). On the side, schisandrol A, by activating the PI3K/AKT pathway, inhibits the IKK/IκBα/NF-κB pathway, reduces neuronal inflammation and oxidative stress, and improves the survival of dopamine neurons in the brain of mice, showing promising prospects in the treatment of PD (Yan et al., 2019). In MPTP-induced PD mice, treatment with high dose of schisandrol A (40 mg/kg) significantly reduce cytokines, such as IL-1β or TNF-α, and inhibit the activity of MDA, but increase SOD, improving antioxidant defences. While low dose of schisandrol A (20 mg/kg) showed no above effect. Additionlly, autophagy-related proteins LC3-II, Beclin1, Parkin, Pink1, and mTOR were expressed after schisandrol A treatment (Zhi et al., 2019).

### Glycosides

Paeoniflorin (PF), a bicyclic monoterpene glycoside (Figure 2), is the main active component of *Paeonia lactiflora* Pall. (family Paeoniaceae, genus *Paeonia*). In addition to anti-inflammation and anti-oxidation, PF also has neuroprotective effects and can be used in the treatment of cerebral ischemia, epilepsy and PD (Gu et al., 2016). Zheng et al. found that PF plays a neuroprotective role through the Bcl-2/Bax/caspase-3 pathway *in vitro* cell experiments. In PD mouse models, PF treatment protected dopaminergic neurons by preventing MPTP-induced declines in the levels of striatal and melanotic dopaminergic transporters (DAT) and tyrosine hydroxylase (TH) proteins and altering dopamine catabolism (Zheng et al., 2017).

### Non-flavonoid Polyphenols

Resveratrol is an antitoxin produced by many plants when they are stimulated. The experiment results showed that resveratrol delay the progression of PD symptoms by activating SIRT1/AKT1 and PI3K/AKT signal pathway (Wang et al., 2018a; Huang et al., 2019). As a compound extracted from *Cannabis sativa* L. (family Cannabaceae, genus *Cannabis*), Cannabidiol (CBD) has a series of physiological functions, such as blocking breast cancer metastasis, treating epilepsy, anti-insomnia and so on. It also has a good effect on the treatment of nervous system diseases. A study suggested that the prevention and protection of CBD on PD seem to be mediated by activating ERK and AKT/mTOR pathways. This protective effect could be eliminated by AKT1/2 inhibitors and mTOR inhibitors (Gugliandolo et al., 2020). In 2020, a Brazilian team published a paper discussing the biological basis for the potential role of CBD, as well as the team’s preclinical and clinical studies of CBD in Parkinson’s disease. The three clinical studies include open label studies (six patients), case series (four patients), and randomized controlled trials (twenty-one patients) (Zuardi et al., 2009; Chagas et al., 2014; Ferreira-Junior et al., 2020). Although these studies have shown beneficial results, they are limited by the small sample size and short follow-up time, which make the results inconclusive. Recently, a first clinical trial of the efficacy of relatively high doses of CBD (20 mg/kg/day) on PD patients examined the tolerability and effectiveness of a range of doses in the PD population (Leehey et al., 2020). However, the study is limited due to the lack of a placebo arm and a small group of participants. And adverse reactions were found at high doses. Therefore, a large number of studies on CDB are still needed to explore the in-depth mechanism of CDB on PD and its biosafety for patients.

### Others

Crocin is a carotenoid found in *Crocus sativus* L. (family Iridaceae, genus *Crocus*), which shows beneficial effects on neurodegenerative diseases through anti-apoptosis, anti-inflammatory and antioxidant activities. However, the exact molecular pathway of the neuroprotective effect of safflower has not been fully elucidated. In rotenone (ROT)-induced rat PD model, crocin visiblystimulated PI3K/AKT pathway and decreased the expression of GSK-3, FoxO3a and downstream caspase-9, presenting a good neuroprotective founction (Salama et al., 2020). Astaxanthin (AST), as a lipid-soluble pigment, inhibits apoptosis and neuronal injury by up-regulating phosphorylation of PI3K and AKT *in vivo* and *vitro*, and it is considered for the treatment of AD and PD (Zarneshan et al., 2020). Moreover, asiatic acid (AA), a pentacyclic triterpene obtained from *Centella asiatica* (L.) Urb. (family Apiaceae, genus *Centella*), has been shown to effectively provide neuroprotection for MPTP-induced neuron cell loss through ERK and PI3K/AKT/mTOR/GSK-3 pathway (Nataraj et al., 2017). The phosphorylation levels of AKT and mTOR could be increased by both Lycium barbarum polysaccharide (LBP) (Wang et al., 2018b) and *Astragalus* polysaccharides (APS) (Tan et al., 2020), effectively alleviate the nigral striatal system degeneration and regulation of cell autophagy, thereby affecting the occurrence and development of PD.

## Discussion

The PI3K/AKT signaling pathway plays an important role in maintaining homeostasis throughout the life cycle. This pathway is triggered by the expression or abnormal regulation of many genes and has been found to be associated with numerous human diseases (Mengqiu et al., 2019). On the one hand, the excessive activation of PI3K/AKT pathway associated with diseases such as cancers and diabetes. On the other hand, deregulating its action might be relevant to cardiovascular diseases, and neurological diseases like AD and PD. Therefore, the study of PI3K/AKT signaling pathway and its upstream and downstream molecular mechanisms is of great significance for the prevention and treatment of various diseases. According to existing studies, many natural compounds have shown symptomatic alleviating effects on neurological diseases AD or PD, and such effects have been found to be related to the regulation of PI3K/AKT signaling pathway. However, it is not certain that whether this pathway plays a key role in this process. In other words, more evidence is needed to determine if this pathway be able to a target for drugs acting on AD or PD. Moreover the complex pathogenesis of these diseases and the multifactorial effects of natural products also bring challenges to explore the synergistic effects of various mechanisms, so that the molecular mechanisms of the neuroprotective effects of natural products also need to be further elucidated.

It is known that numerous natural products with medicinal potential are derived from a wide variety of vegetables, fruits and Chinese herbs, some scholars believe that dietary adjustments might be help for the prevention and treatment of diseases. (Kitagishi et al., 2014). For instance, multiple clinical trials have investigated the effect of curcumin on AD. But in most of these trials, curcumin was used as a dietary supplement, like an ongoing clinical trial in India about the efficacy and safety of curcumin formulation in Alzheimer’s disease (Bhat et al., 2019). Even though there are no clinical reports of true dietary supplements for the treatment of neurological diseases, preclinical and clinical studies have demonstrated in some respects that diet still has merit as an adjunct preventive approach.

In reviewing the literature, we found that a variety of common natural polyphenols possess neuroprotective effects, including flavonoids and non-flavonoids, such as curcumin, icariin, baicalein and cannabidiol. However, it is a pity that many natural compounds, including natural flavonoids, are often limited in their application due to bioavailability and other defects. Consequently, using natural products as lead compounds and key pharmacophore targets to develop more effective and safer derivatives with high bioavailability may be a promising strategy for new drug development. For instance, curcumin is eliminated *in vivo* easily as its hydrophobicity and low bioavailability (Gupta et al., 2012; De Oliveira et al., 2016; Mirzaei et al., 2017), but such problems can be solved by modifying the structure of curcumin and synthesizing a series of derivatives (Bagheri et al., 2020). And the low solubility of baicalein is able to improve by nano-loading, that is, the oral bioavailability of baicalein is improved by nano-emulsion or nano-crystal loaded with baicalein (Liu et al., 2016; Yin et al., 2017). We summarize the natural products mentioned above that have the potential to treat AD and PD, as shown in Supplementary Figures S1 and S2. The structure of the active ingredients is show in Figure 1 and Figure 2, and so do some relevant information for reference (Table 1). It can be learned that the treatment of natural products is a relatively long process, and some natural products could exert better effects at lower doses. But some are ineffective at low doses and require high concentrations to be effective. The therapeutic effects are connected with the dosage, time, and mode of administration. For instance, at low concentrations (0.12–16 µM), BBR dramatically recedes 6-OHDAinduced cytotoxicity through the activation of PI3K/AKT/Bcl-2 cell survival and Nrf2/HO-1 antioxidative signaling pathways. However, However, treatment with high concentrations (16 µM) BBR did not show protective effect (Zhang et al., 2017). A study investigated the role of curcumin and erythropoietin in an ICV-STZ rat model (a model for sporadic dementia of the Alzheimer’s type (SDAT)) (Samy et al., 2016). The experimental animals were given vehicle, oral administration of curcumin (80 mg/kg/day), an intraperitoneal injection of erythropoietin (500 IU/kg every other day) as well as combined curcumin and erythropoietin for 3 months. The results showed that curcumin and/or erythropoietin were beneficial in restoring the behavioral, histological, and biochemical changes induced by ICV-STZ. And the long-term adverse effects of curcumin were lower than those of erythropoietin. In contrast, Bassani et al. found no beneficial effect in short-term spatial memory in ICV-STZ rats by evaluating extended oral curcumin (doses of 25, 50, and 100 mg/kg) (Bassani et al., 2017). But improvements were found in short-term cognitive memory. In addition, the combination of natural products with existing drugs is also a direction worth exploring. A report indicates that combination treatment of icariin and L-3,4-dihydroxyphenylalanine (l-DOPA) are beneficial for the treatment of dopamine neurotoxicity in 6-OHDA injury (Lu et al., 2018).

Each new drug should be clinically tested after developed but before used in humans. Practically, the fact that the results of animal and human are not exactly consistent is worrisome. In animal studies, certain natural products have shown great promise in treating Alzheimer’s disease and cognitive aging, but this has not been observed in all clinical trials. After all, there is no animal model at the moment really reproduces all the symptoms observed in human pathology. In contrast to animal studies, the number of confirmed human studies is limited, and many of the results remain controversial. However, natural products are widely available, relatively safe and easy to obtain, making the research still extraordinary significance. Consequently, it is essential to conduct more long-term studies to assess the possibility of side effects from chronic administration in humans.

## Prospect

Scientists have never stopped exploring the complex pathogenesis of AD and PD. It is exciting that great progress has been made in recent years, which supply more possibilities to overcome these diseases. However, we also note that a small number of new drugs have been developed for the treatment of AD and PD over the years, and most of them are still in the stage of basic research. The pathological changes associated with the onset of neurodegenerative diseases are irreversible. When cognitive impairment shows up in patients, the course of the disease are often in the middle or late stage, as a result, the treatment can only slow down rather than reverse the development of the disease. Undoubtedly, it is particularly important for early prevention and diagnosis of neurodegenerative diseases. Further research into its pathogenesis is needed to develop drugs to reverse the progression of AD and PD. Substantial amounts of studies on natural products with multiple activities have proved to be of positive significance for the treatment of such diseases. In-depth research on natural products will bring more hope for the treatment of diseases, and at the same time promote the development of traditional Chinese herbal medicine. The direction for the continued research of natural products has also been proposed in the previous discussion: (I) the diseases prevention and control mechanisms of natural products are worthy of further investigation; (II) structural modification as a lead compound; (III) application of nanotechnology for decoration; (IV) combination with other drugs. Finally, it should be noted that the key to determining the safety and effectiveness of the final drug lies in conduct extensive clinical trials.

## Glossary

6-OHDA: 6-hydroxydopamine
AA: asiatic acid
AD: Alzheimer’s disease
AF: amentoflavone
AKT: protein kinase B
AMPA: α-amino-3-hydroxy-5-methyl-4-isoxazolpropionic acid
APP: amyloid precursor protein
APS: astragalus polysaccharides
ARE: antioxidant response element
AST: astaxanthin
Aβ,: amyloid protein
BACE1: β-secretase 1
Bad: Bcl2-antagonist of cell death
Bax: BCL2-associated X protein
Bcl-2: B-cell lymphoma 2
BDNF: Brain-derived neurotrophic factor
BIM: Bcl-2-like protein 11
CBD: Cannabidiol
CNKI: China national knowledge infrastructure
CREB: cAMP response element-binding protein
DA: Dopaminergic
DAT: Dopaminergic transporters
DHM: Dihydromyricetin
DNA-PK: DNA-dependent protein kinases
ERK: Extracellular signal-regulated kinase
FB: Fructus broussonetiae
FGF: Fibroblast growth factor
FoxO1: Caspase-9 and forkhead box protein
GLUT4: Glucose transporter 4
GPCR: G protein coupled receptors
GSK-3β: Glycogen synthase kinase-3β
HO-1: Heme oxygenase 1
Hsp: Heat shock protein
ICA: Icariin
IFN-α: Interferon-α; α TrkB, tyrosine kinase B
IGF: Insulin-like growth factor
IKK: IκB kinase
IL1β: Interleukin-1 β
ILK: Integrin-linked kinase
IRS: Insulin receptor substrate
JNK: c-Jun NH(2)-terminal kinase
LBP: Lycium barbarum polysaccharide
l-DOPA: L-3,4-dihydroxyphenylalanine
LSF: Active fraction of lychee seed
LTP: Long term potentiation
MAO: Monoamine oxidase
mLST8: SEC13 protein 8
MMP: Mitochondrial membrane potential
MMP+: 1-methyl-4-phenyl-pyridine
MPTP: 1-methyl-4-phenyl-1,2,3,6-tetrahydropyridine
mTOR: Mammalian target of rapamycin
mTORC2: Mechanistic target of rapamycin complex 2
NE: Norepinephrine
NFT: Neurofibrillary tangles
NF-κB: Nuclear factor kappa-light-chain-enhancer of activated B cells
NMDA: N-methyl-d-aspartic acid
Nrf2: Nuclear factor erythrocyte 2-related factor 2
PARP: Poly ADP-ribose polymerase
PC: Procyanidine
PC12: Pheochromocytoma 12
PD: Parkinson’s disease
PDGF: Platelet-derived growth factor
PDK1: Phosphatidylinositol dependent protein kinase 1
PF: Paeoniflorin
PHLPP: PH domain and leucine rich repeat protein phosphatase
PI3K: Phosphoinositide 3-kinase
PIP2: Phosphatidylinositol (4,5)-disphosphate
PIP3: Phosphatidylinositol (3,4,5)-trisphosphate
PKB: Protein kinase B
PKC: Protein kinase C
PP2A: Protein phosphatase 2 A
PS: Pterostilbene
PT: Piceatannol
PTEN: Phosphatase and tensin homolog
PUE: Puerarin
REDD1: DNA damage responses 1
ROS: Rwactive oxygen species
ROT: Rotenone
RTK: Receptor tyrosine kinase
Sal: Salidroside
SchA: Schizandrol A
SCL: Schisandra chinensis lignans
SDAT: Sporadic dementia of the Alzheimer’s type
SH: Src Homology
SHIP1: Src homology domain-containing inositol 5′-phosphatase 1
SIRT1: Silent mating type information regulation 2 homolog-1
SN: Substantia nigra
SNpc: Substantia nigra compacta
SP: Senile plaque
TA: Tovophyllin A
t-BHP|: Tert-butyl peroxide
TH: Tyrosine hydroxylase
TNF-α: Tumor necrosis factor-α
VOZ: Zanthoxylum bungeanum volatile oil extract
VPS34: Vacuolar protein sorting 34
WEZ: Zanthoxylum bungeanum water extract

## References

[B1] Altomare D. A. Testa J. R. Testa A. Joseph R. (2005). Perturbations of the AKT signaling pathway in human cancer. Oncogene. 24 (50), 7455–7464. 10.1038/sj.onc.1209085 16288292

[B2] Bagheri H. Ghasemi F. Barreto G. E. Rafiee R. Sathyapalan T. Sahebkar A. (2020). Effects of curcumin on mitochondria in neurodegenerative diseases. Biofactors. 46 (1), 5–20. 10.1002/biof.1566 31580521

[B3] Bassani T. B. Turnes J. M. Moura E. L. R. Bonato J. M. Cóppola-Segovia V. Zanata S. M. (2017). Effects of curcumin on short-term spatial and recognition memory, adult neurogenesis and neuroinflammation in a streptozotocin-induced rat model of dementia of Alzheimer’s type. Behav. Brain Res. 335, 41–54. 10.1016/j.bbr.2017.08.014 28801114

[B4] Batista P. Pereira A. (2016). Quality of life in patients with neurodegenerative diseases. J. Neurol. Neurosci. 7 (1). 10.21767/2171-6625.100074

[B5] Bellacosa A. Kumar C. C. Cristofano A. D. Testa J. R. (2005). Activation of AKT kinases in cancer: implications for therapeutic targeting. Adv. Cancer Res. 94 (1), 29–86. 10.1016/S0065-230X(05)94002-5 16095999

[B6] Bertacchini J. Heidari N. Mediani L. Capitani S. Shahjahani M. Ahmadzadeh A. (2015). Targeting PI3K/AKT/mTOR network for treatment of leukemia. Cell. Mol. Life Sci. 72 (12), 2337–2347. 10.1007/s00018-015-1867-5 25712020 PMC11113278

[B7] Bhat A. Mahalakshmi A. M. Ray B. Tuladhar S. Hediyal T. A. Manthiannem E. (2019). Benefits of curcumin in brain disorders. Biofactors 45 (5), 666–689. 10.1002/biof.1533 31185140

[B8] Cao Q. Qin L. Huang F. Wang X. Yang L. Shi H. (2017). Amentoflavone protects dopaminergic neurons in MPTP-induced Parkinson’s disease model mice through PI3K/Akt and ERK signaling pathways. Toxicol. Appl. Pharmacol. 319, 80–90. 10.1016/j.taap.2017.01.019 28185818

[B9] Chagas M. H. N. Zuardi A. W. Tumas V. Pena-Pereira M. A. Sobreira E. T. Bergamaschi M. M. (2014). Effects of cannabidiol in the treatment of patients with Parkinson’s disease: an exploratory double-blind trial. J. Psychopharmacol. 28 (11), 1088–1098. 10.1177/0269881114550355 25237116

[B10] Chen W.-F. Wu L. Du Z.-R. Chen L. Xu A.-L. Chen X.-H. (2017). Neuroprotective properties of icariin in MPTP-induced mouse model of Parkinson’s disease: involvement of PI3K/Akt and MEK/ERK signaling pathways. Phytomedicine. 25, 93–99. 10.1016/j.phymed.2016.12.017 28190476

[B11] Cheng Q. Zhang S. Ding F. (2012). Determination of salidroside in rats and its pharmacokinetic profile. Lishizhen Med. Materia Med. Res. 10, 2422–2424.

[B12] Chong Z. Z. Shang Y. C. Wang S. Maiese K. (2012). A critical kinase cascade in neurological disorders: PI3K, Akt and mTOR. Future Neurol. 7 (6), 733–748. 10.2217/fnl.12.72 23144589 PMC3491909

[B13] Coria H. M. Mendoza Rojas M. X. Arrieta Cruz I. Valdés H. E. L. (2019). Preclinical research of dihydromyricetin for brain aging and neurodegenerative diseases. Front. Pharmacol. 10, 1334. 10.3389/fphar.2019.01334 31780947 PMC6859532

[B14] Cox K. H. Pipingas A. Scholey A. B. (2015). Investigation of the effects of solid lipid curcumin on cognition and mood in a healthy older population. J. Psychopharmacol. 29 (5), 642–651. 10.1177/0269881114552744 25277322

[B15] Cui J. Shan R. Cao Y. Zhou Y. Liu C. Fan Y. (2021). Protective effects of ginsenoside Rg2 against memory impairment and neuronal death induced by Aβ25-35 in rats. J. Ethnopharmacology 266, 113466. 10.1016/j.jep.2020.113466 33049344

[B16] Cui J. Wang J. Zheng M. Gou D. Zhou Y. (2017a). Ginsenoside Rg2 protects PC12 cells against β-amyloid 25-35 -induced apoptosis via the phosphoinositide 3-kinase/Akt pathway. Chem. Biol. Interact. 275, 152-161. 10.1016/j.cbi.2017.07.021 28756148

[B17] Cui W. Wang S. Wang Z. Wang Z. Sun C. Zhang Y. (2017b). Inhibition of PTEN attenuates endoplasmic reticulum stress and apoptosis via activation of PI3K/AKT pathway in Alzheimer’s disease. Neurochem. Res. 42 (11), 3052–3060. 10.1007/s11064-017-2338-1 28819903

[B18] Cui Q. Li X. Zhu H. (2016). Curcumin ameliorates dopaminergic neuronal oxidative damage via activation of the Akt/Nrf2 pathway. Mol. Med. Rep. 13 (2), 1381–1388. 10.3892/mmr.2015.4657 26648392

[B19] De Oliveira M. R. Jardim F. R. Setzer W. N. Nabavi S. M. Nabavi S. F. (2016). Curcumin, mitochondrial biogenesis, and mitophagy: exploring recent data and indicating future needs. Biotechnol. Adv. 34, 813–826. 10.1016/j.biotechadv.2016.04.004 27143655

[B20] Deng H. Jia Y. Pan D. Ma Z. (2020). Berberine alleviates rotenone-induced cytotoxicity by antioxidation and activation of PI3K/Akt signaling pathway in SH-SY5Y cells. Neuroreport. 31 (1), 41–47. 10.1097/WNR.0000000000001365 31688419

[B21] Deng S. Rong H. Tu H. Zheng B. Mu X. Zhu L. (2019). Molecular basis of neurophysiological and antioxidant roles of Szechuan pepper. Biomed. Pharmacother. 112, 108696. 10.1016/j.biopha.2019.108696 30818139

[B22] Do T. D. Economou N. J. Chamas A. Buratto S. K. Shea J.-E. Bowers M. T. (2014). Interactions between amyloid-β and tau fragments promote aberrant aggregates: implications for amyloid toxicity. J. Phys. Chem. B. 118 (38), 11220–11230. 10.1021/jp506258g 25153942 PMC4174992

[B23] Dobbin Z. Landen C. (2013). The importance of the PI3K/AKT/MTOR pathway in the progression of ovarian cancer. Int J Mol Sci. 14 (4), 8213–8227. 10.3390/ijms14048213 23591839 PMC3645739

[B24] Fan F. Yang L. Li R. Zou X. Li N. Meng X. (2020). Salidroside as a potential neuroprotective agent for ischemic stroke: a review of sources, pharmacokinetics, mechanism and safety. Biomed. Pharmacother. 129, 110458. 10.1016/j.biopha.2020.110458 32603893

[B25] Ferreira-Junior N. C. Campos A. C. Guimarães F. S. Del-Bel E. Zimmermann P. M. d. R. Brum Junior L. (2020). Biological bases for a possible effect of cannabidiol in Parkinson’s disease. Braz. J. Psychiatry 42 (2), 218–224. 10.1590/1516-4446-2019-0460 31314869 PMC7115443

[B26] Fu W. Zhuang W. Zhou S. Wang X. (2015). Plant-derived neuroprotective agents in Parkinson’s disease. Am. J. Transl Res. 7 (7), 1189–1202. 26328004 PMC4548312

[B27] Fu Z. Yang J. Wei Y. Li J. (2016). Effects of piceatannol and pterostilbene against β-amyloid-induced apoptosis on the PI3K/Akt/Bad signaling pathway in PC12 cells. Food Funct. 7 (2), 1014–1023. 10.1039/c5fo01124h 26757883

[B28] Gao L. Zhou F. Wang K.-x. Zhou Y.-z. Du G.-h. Qin X.-m. (2020). Baicalein protects PC12 cells from Aβ25-35-induced cytotoxicity via inhibition of apoptosis and metabolic disorders. Life Sci. 248, 117471. 10.1016/j.lfs.2020.117471 32112868

[B29] Gao Q. Yang M. Zuo Z. (2018). Overview of the anti-inflammatory effects, pharmacokinetic properties and clinical efficacies of arctigenin and arctiin from Arctium lappa L. Acta Pharmacol. Sin. 39 (5), 787–801. 10.1038/aps.2018.32 29698388 PMC5943914

[B30] Garofalo R. S. Orena S. J. Rafidi K. Torchia A. J. Stock J. L. Hildebrandt A. L. (2003). Severe diabetes, age-dependent loss of adipose tissue, and mild growth deficiency in mice lacking Akt2/PKBβ. J. Clin. Invest. 112 (2), 197–208. 10.1172/jci16885 12843127 PMC164287

[B31] Ghoneum A. Said N. (2019). PI3K-AKT-mTOR and NFκB pathways in ovarian cancer: implications for targeted therapeutics. Cancers 11 (7), 949. 10.3390/cancers11070949 PMC6679095 31284467

[B32] Gong J. Zhang L. Zhang Q. Li X. Xia X.-J. Liu Y.-Y. (2018). Lentiviral vector-mediated SHC3 silencing exacerbates oxidative stress injury in nigral dopamine neurons by regulating the PI3K-AKT-FoxO signaling pathway in rats with Parkinson’s disease. Cell. Physiol. Biochem. 49 (3), 971–984. 10.1159/000493228 30184529

[B33] Gu X.-S. Wang F. Zhang C.-Y. Mao C.-J. Yang J. Yang Y.-P. (2016). Neuroprotective effects of paeoniflorin on 6-OHDA-lesioned rat model of Parkinson’s disease. Neurochem. Res. 41 (11), 2923–2936. 10.1007/s11064-016-2011-0 27447883

[B34] Gugliandolo A. Pollastro F. Bramanti P. Mazzon E. (2020). Cannabidiol exerts protective effects in an *in vitro* model of Parkinson’s disease activating AKT/mTOR pathway. Fitoterapia 143, 104553. 10.1016/j.fitote.2020.104553 32184097

[B35] Guo C.-h. Cao T. Zheng L.-t. Waddington J. L. Zhen X.-c. (2020). Development and characterization of an inducible Dicer conditional knockout mouse model of Parkinson’s disease: validation of the antiparkinsonian effects of a sigma-1 receptor agonist and dihydromyricetin. Acta Pharmacol. Sin. 41 (4), 499–507. 10.1038/s41401-020-0379-5 32112040 PMC7468551

[B36] Gupta S. C. Patchva S. Koh W. Aggarwal B. B. (2012). Discovery of curcumin, a component of golden spice, and its miraculous biological activities. Clin. Exp. Pharmacol. Physiol. 39 (3), 283–299. 10.1111/j.1440-1681.2011.05648.x 22118895 PMC3288651

[B37] Hanada M. Feng J. Hemmings B. A. (2004). Structure, regulation and function of PKB/AKT--a major therapeutic target. Biochim. Biophys. Acta 1697 (1), 3–16. 10.1016/j.bbapap.2003.11.009 15023346

[B38] Hetman M. Chen W. T. Kuo Y. Y. Lin G. B. Lu C. H. Hsu H. P. (2020). Thermal cycling protects SH-SY5Y cells against hydrogen peroxide and β-amyloid-induced cell injury through stress response mechanisms involving Akt pathway. PLoS One 15 (10), e0240022. 10.1371/journal.pone.0240022 33002038 PMC7529293

[B39] Hu M. Li F. Wang W. (2018). Vitexin protects dopaminergic neurons in MPTP-induced Parkinson’s disease through PI3K/Akt signaling pathway. Drug Des. Devel. Ther. 12, 565–573. 10.2147/dddt.s156920 PMC5859909 29588573

[B40] Hu T. Li S. Liang W.-Q. Li S.-S. Lu M.-N. Chen B. (2020). Notoginsenoside R1-induced neuronal repair in models of alzheimer disease is associated with an alteration in neuronal hyperexcitability, which is regulated by nav. Front. Cel. Neurosci. 14, 280. 10.3389/fncel.2020.00280 PMC7500285 33088260

[B41] Huang N. Zhang Y. Chen M. Jin H. Nie J. Luo Y. (2019). Resveratrol delays 6-hydroxydopamine-induced apoptosis by activating the PI3K/Akt signaling pathway. Exp. Gerontol. 124, 110653. 10.1016/j.exger.2019.110653 31295526

[B42] Huang Y. Sun L. Zhu S. Xu L. Liu S. Yuan C. (2020). Neuroprotection against Parkinson’s disease through the activation of akt/gsk3β signaling pathway by Tovophyllin A. Front. Neurosci. 14, 723. 10.3389/fnins.2020.00723 32742256 PMC7364155

[B43] Jiang B. Le L. Pan H. Hu K. Xu L. Xiao P. (2014a). Dihydromyricetin ameliorates the oxidative stress response induced by methylglyoxal via the AMPK/GLUT4 signaling pathway in PC12 cells. Brain Res. Bull. 109, 117–126. 10.1016/j.brainresbull.2014.10.010 25451453

[B44] Jiang E. P. Wang S. Q. Wang Z. Yu C. R. Chen J. G. Yu C. Y. (2014b). [Effect of Schisandra chinensis lignans on neuronal apoptosis and p-AKT expression of rats in cerebral ischemia injury model]. Zhongguo Zhong Yao Za Zhi 39 (9), 1680–1684. 10.4268/cjcmm20140927 25095384

[B45] Kandezi N. Mohammadi M. Ghaffari M. Gholami M. Motaghinejad M. Safari S. (2020). Novel insight to neuroprotective potential of curcumin: a mechanistic review of possible involvement of mitochondrial biogenesis and PI3/akt/GSK3 or PI3/akt/CREB/BDNF signaling pathways. Int. J. Mol. Cel Med. 9 (1), 1–32. 10.22088/IJMCM.BUMS.9.1.1 PMC7422850 32832482

[B46] Kitagishi Y. Nakanishi A. Ogura Y. Matsuda S. (2014). Dietary regulation of PI3K/AKT/GSK-3β pathway in Alzheimer’s disease. Alzheimers Res. Ther. 6 (3), 35. 10.1186/alzrt265 25031641 PMC4075129

[B47] Kou X. Li J. Bian J. Yang Y. Yang X. Fan J. (2015). Ampelopsin attenuates 6-OHDA-induced neurotoxicity by regulating GSK-3β/NRF2/ARE signalling. J. Funct. Foods. 19, 765–774. 10.1016/j.jff.2015.10.010

[B48] Kou X. Liu X. Chen X. Li J. Yang X. Fan J. (2016). Ampelopsin attenuates brain aging of D-gal-induced rats through miR-34a-mediated SIRT1/mTOR signal pathway. Oncotarget. 7 (46), 74484–74495. 10.18632/oncotarget.12811 27780933 PMC5342681

[B49] Kou X. Shen K. An Y. Qi S. Dai W.-X. Yin Z. (2012). Ampelopsin inhibits H2 O2 -induced apoptosis by ERK and akt signaling pathways and up-regulation of heme oxygenase-1. Phytother. Res. 26 (7), 988–994. 10.1002/ptr.3671 22144097

[B50] Laplante M. Sabatini D. M. (2012). mTOR signaling in growth control and disease. Cell 149 (2), 274–293. 10.1016/j.cell.2012.03.017 22500797 PMC3331679

[B51] Leehey M. A. Liu Y. Hart F. Epstein C. Cook M. Sillau S. (2020). Safety and tolerability of cannabidiol in Parkinson disease: an open label, dose-escalation study. Cannabis Cannabinoid Res. 5 (4), 326–336. 10.1089/can.2019.0068 33381646 PMC7759259

[B52] Li H. Kang T. Qi B. Kong L. Jiao Y. Cao Y. (2016). Neuroprotective effects of ginseng protein on PI3K/Akt signaling pathway in the hippocampus of D -galactose/AlCl 3 inducing rats model of Alzheimer’s disease. J. Ethnopharmacology 179, 162–169. 10.1016/j.jep.2015.12.020 26721223

[B53] Li Q. Wang J. Li Y. Xu X. (2018). Neuroprotective effects of salidroside administration in a mouse model of Alzheimer’s disease. Mol. Med. Rep. 17, 7287–7292. 10.3892/mmr.2018.8757 29568861

[B54] Li, Y.-h. Jin Y. Wang X.-s. Chen X.-l. Chen H.-b. Xu J. (2020). Neuroprotective effect of fructus broussonetiae on APP/PS1 mice via upregulation of AKT/β-Catenin signaling. Chin. J. Integr. Med. 27, 115. 10.1007/s11655-019-3178-4 31903532

[B55] Li Y. Zhao J. Hölscher C. (2017). Therapeutic potential of baicalein in Alzheimer’s disease and Parkinson’s disease. CNS Drugs. 31 (8), 639–652. 10.1007/s40263-017-0451-y 28634902

[B56] Li, Z. Jiang T. Lu Q. Xu K. He J. Xie L. (2020). Berberine attenuated the cytotoxicity induced by t-BHP via inhibiting oxidative stress and mitochondria dysfunction in PC-12 cells. Cell. Mol. Neurobiol. 40 (4), 587–602. 10.1007/s10571-019-00756-7 31828466 PMC11448801

[B57] Liang J. Wu Y. Yuan H. Yang Y. Xiong Q. Liang C. (2019). Dendrobium officinale polysaccharides attenuate learning and memory disabilities via anti-oxidant and anti-inflammatory actions. Int. J. Biol. Macromolecules. 126, 414–426. 10.1016/j.ijbiomac.2018.12.230 30593810

[B58] Liao S. F. Wang H. T. Yan F. X. Zheng Y. X. Zeng Z. W. Zheng W. H. (2014). [Protective effect and mechanisms of dihydromyricetin on PC12 cells induced by oxidative injury]. Zhong Yao Cai 37 (6), 1014–1020. 25470969

[B59] Liu W. Zhai Y. Heng X. Che F. Y. Chen W. Sun D. (2016). Oral bioavailability of curcumin: problems and advancements. J. Drug Target. 24 (8), 694–702. 10.3109/1061186x.2016.1157883 26942997

[B60] Zhu Q. Zhuang X. Lu J. (2019). Neuroprotective effects of baicalein in animal models of Parkinson’s disease: a systematic review of experimental studies. Phytomedicine. 55, 302–309. 10.1016/j.phymed.2018.09.215 30385133

[B61] Liying Y. Hongyan W. Lijun L. Anmu X. (2018). The role of insulin/IGF-1/PI3K/Akt/GSK3β signaling in Parkinson’s disease dementia. Front. Neurosci. 12, 73. 10.3389/fnins.2018.00073 29515352 PMC5826217

[B62] Lu D.-S. Chen C. Zheng Y.-X. Li D.-D. Wang G.-Q. Liu J. (2018). Combination treatment of icariin and L-DOPA against 6-OHDA-lesioned dopamine neurotoxicity. Front. Mol. Neurosci. 11, 155. 10.3389/fnmol.2018.00155 29867347 PMC5964195

[B63] Lu Q. Zhu H. Liu X. Tang C. (2020). Icariin sustains the proliferation and differentiation of Aβ25-35-treated hippocampal neural stem cells via the BDNF-TrkB-ERK/Akt signaling pathway. Neurol. Res. 42 (11), 936–945. 10.1080/01616412.2020.1792701 32727295

[B64] Lulin N. Junxia X. Honglian L. Zaijun Z. Ying Y. Xinfeng H. (2017). Ginsenoside Rg1 ameliorates behavioral abnormalities and modulates the hippocampal proteomic change in triple transgenic mice of Alzheimer’s disease. Oxid Med. Cel Longev. 2017, 1–17. 10.1155/2017/6473506 PMC5674513 29204248

[B65] Luo S. Kang S. S. Wang Z.-H. Liu X. Day J. X. Wu Z. (2019). Akt phosphorylates NQO1 and triggers its degradation, abolishing its antioxidative activities in Parkinson’s disease. J. Neurosci. 39 (37), 7291–7305. 10.1523/JNEUROSCI.0625-19.2019 31358653 PMC6759025

[B66] Ma B. Meng X. Wang J. Sun J. Ren X. Qin M. (2014). Notoginsenoside R1 attenuates amyloid-β-induced damage in neurons by inhibiting reactive oxygen species and modulating MAPK activation. Int. Immunopharmacology. 22(1), 151–159. 10.1016/j.intimp.2014.06.018 24975829

[B67] Ma X.-W. Guo R.-Y. (2017). Dose-dependent effect of Curcuma longa for the treatment of Parkinson’s disease. Exp. Ther. Med. 13 (5), 1799–1805. 10.3892/etm.2017.4225 28565770 PMC5443238

[B68] Mario P. M. Jorge M. F. Cesar R. T. Mcarmen R. T. (2016). Curcumin and health. Molecules 21 (3), 264. 10.3390/molecules21030264 26927041 PMC6273481

[B69] Martins A. C. Morcillo P. Ijomone O. M. Venkataramani V. Harrison F. E. Lee E. (2019). New insights on the role of manganese in Alzheimer’s disease and Parkinson’s disease. Int J Environ Res Public Health. 16 (19), 3546. 10.3390/ijerph16193546 PMC6801377 31546716

[B70] Matsuda S. Ikeda Y. Murakami M. Nakagawa Y. Tsuji A. Kitagishi Y. (2019). Roles of PI3K/AKT/GSK3 pathway involved in psychiatric illnesses. Diseases. 7 (1), 22. 10.3390/diseases7010022 PMC6473240 30781836

[B71] Matsuo F. S. Andrade M. F. Loyola A. M. da Silva S. J. Silva M. J. B. Cardoso S. V. (2018). Pathologic significance of AKT, mTOR, and GSK3β proteins in oral squamous cell carcinoma-affected patients. Virchows Arch. 472, 983. 10.1007/s00428-018-2318-0 29713826

[B72] Memmott R. M. Dennis P. A. (2009). Akt-dependent and -independent mechanisms of mTOR regulation in cancer. Cell Signal. 21 (5), 656–664. 10.1016/j.cellsig.2009.01.004 19166931 PMC2650010

[B73] Meng X. Sun G. Ye J. Xu H. Wang H. Sun X. (2014). Notoginsenoside R1-mediated neuroprotection involves estrogen receptor-dependent crosstalk between Akt and ERK1/2 pathways: a novel mechanism of Nrf2/ARE signaling activation. Free Radic. Res. 48 (4), 445. 10.3109/10715762.2014.885117 24437944

[B74] Mengqiu S. Ann B. Zigang D. Mee-Hyun L (2019). AKT as a therapeutic target for cancer. Cancer Res. 79, 1019. 10.1158/0008-5472.CAN-18-2738 30808672

[B75] Ming H. Fangming L. Weidong W. (2018). Vitexin protects dopaminergic neurons in MPTP-induced Parkinson’s disease through PI3K/Akt signaling pathway. Drug Des. Dev. Ther. 12, 565–573. 10.2147/DDDT.S156920 PMC5859909 29588573

[B76] Mirdamadi Y. Bommhardt U. Goihl A. Guttek K. Zouboulis C. C. Quist S. 2017). Insulin and Insulin-like growth factor-1 can activate the phosphoinositide-3-kinase/Akt/FoxO1 pathway in T cells *in vitro* . Dermato-Endocrinology 9(1), e1356518. 10.1080/19381980.2017.1356518 29484090 PMC5821168

[B77] Mirzaei H. Shakeri A. Rashidi B. Jalili A. Banikazemi Z. Sahebkar A. (2017). Phytosomal curcumin: a review of pharmacokinetic, experimental and clinical studies. Biomed. Pharmacother. 85, 102–112. 10.1016/j.biopha.2016.11.098 27930973

[B78] Mu S. Li Y. Liu B. Wang W. Chen S. Wu J. (2016). Dihydromyricetin ameliorates 3NP-induced behavioral deficits and striatal injury in rats. J. Mol. Neurosci. 60, 267. 10.1007/s12031-016-0801-0 27501707

[B79] Nataraj J. Manivasagam T. Justin Thenmozhi A. Essa M. M. (2017). Neurotrophic effect of asiatic acid, a triterpene of *Centella asiatica* against chronic 1-methyl 4-phenyl 1, 2, 3, 6-tetrahydropyridine hydrochloride/probenecid mouse model of Parkinson’s disease: the role of MAPK, PI3K-Akt-GSK3β and mTOR signalling pathways. Neurochem. Res. 42 (5), 1354–1365. 10.1007/s11064-017-2183-2 28181071

[B80] Parkinson G. T. Hanley J. G. (2018). Mechanisms of AMPA receptor endosomal sorting. Front. Mol. Neurosci. 11. 10.3389/fnmol.2018.00440 PMC6289981 30568574

[B81] Petrik L. Santry L. Moorehead R. Jücker M. Petrik J. (2016). Akt isoform specific effects in ovarian cancer progression. Oncotarget 7 (46), 74820-74833. 10.18632/oncotarget.11204 27533079 PMC5342704

[B82] Qi Y. Cheng X. Gong G. Yan T. Du Y. Wu B. (2020). Synergistic neuroprotective effect of schisandrin and nootkatone on regulating inflammation, apoptosis and autophagy via the PI3K/AKT pathway. Food Funct. 11 (3), 2427–2438. 10.1039/c9fo02927c 32129354

[B83] Qi Y. Dou D.-Q. Jiang H. Zhang B.-B. Qin W.-Y. Kang K. (2017). Arctigenin attenuates learning and memory deficits through PI3k/akt/GSK-3β pathway reducing tau hyperphosphorylation in aβ-induced AD mice. Planta Med. 83 (1–2), 51–56. 10.1055/s-0042-107471 27224270

[B84] Ringman J. M. Frautschy S. A. Teng E. Begum A. N. Bardens J. Beigi M. (2012). Oral curcumin for Alzheimer’s disease: tolerability and efficacy in a 24-week randomized, double blind, placebo-controlled study. Alzheimers Res. Ther. 4 (5), 43. 10.1186/alzrt146 23107780 PMC3580400

[B85] Salama R. M. Abdel-Latif G. A. Abbas S. S. El Magdoub H. M. Schaalan M. F. (2020). Neuroprotective effect of crocin against rotenone-induced Parkinson’s disease in rats: interplay between PI3K/Akt/mTOR signaling pathway and enhanced expression of miRNA-7 and miRNA-221. Neuropharmacology 164, 107900. 10.1016/j.neuropharm.2019.107900 31811872

[B86] Samy D. M. Ismail C. A. Nassra R. A. Zeitoun T. M. Nomair A. M. (2016). Downstream modulation of extrinsic apoptotic pathway in streptozotocin-induced Alzheimer’s dementia in rats: erythropoietin versus curcumin. Eur. J. Pharmacol. 770, 52–60. 10.1016/j.ejphar.2015.11.046 26638997

[B87] Satoru M. Yukie N. Ai T. Yasuko K. Atsuko N. Toshiyuki M. (2018). Implications of PI3K/AKT/PTEN signaling on superoxide dismutases expression and in the pathogenesis of Alzheimer’s disease. Diseases 6 (2), 28 10.3390/diseases6020028 PMC6023281 29677102

[B88] Shane M. Aggleton J. P. (2019). Space and memory (far) beyond the *Hippocampus*: many subcortical structures also support cognitive mapping and mnemonic processing. Front. Neural Circu. 13, 52. 10.3389/fncir.2019.00052 PMC6692652 31447653

[B89] Shanu M. Komal J. (2015). Ganetespib: research and clinical development. Oncotargets Ther. 8, 1849. 10.2147/OTT.S65804 PMC4521669 26244021

[B90] Shi C. Zheng D.-d. Fang L. Wu F. Kwong W. H. Xu J. (2012). Ginsenoside Rg1 promotes nonamyloidgenic cleavage of APP via estrogen receptor signaling to MAPK/ERK and PI3K/Akt. Biochim Biophys Acta. 1820 (4), 453–460. 10.1016/j.bbagen.2011.12.005 22178929

[B91] Small G. W. Siddarth P. Li Z. Miller K. J. Ercoli L. Emerson N. D. (2018). Memory and brain amyloid and tau effects of a bioavailable form of curcumin in non-demented adults: a double-blind, placebo-controlled 18-month trial. Am. J. Geriatr. Psychiatry 26 (3), 266–277. 10.1016/j.jagp.2017.10.010 29246725

[B92] Soliman G. (2013). The role of mechanistic target of rapamycin (mTOR) complexes signaling in the immune responses. Nutrients 5 (6), 2231–2257. 10.3390/nu5062231 23783557 PMC3725503

[B93] Song L. Yao L. Zhang L. Piao Z. Lu Y. (2020). Schizandrol A protects against Aβ1-42-induced autophagy via activation of PI3K/AKT/mTOR pathway in SH-SY5Y cells and primary hippocampal neurons. Naunyn-schmiedeberg’s Arch. Pharmacol. 393 (9), 1739–1752. 10.1007/s00210-019-01792-2 31900522

[B94] Spinelli M. Fusco S. Grassi C. (2019). Brain insulin resistance and hippocampal plasticity: mechanisms and biomarkers of cognitive decline. Front. Neurosci. 13, 788. 10.3389/fnins.2019.00788 31417349 PMC6685093

[B95] Sugiyama M. G. Fairn G. D. Antonescu C. N. (2019). Akt-ing up just about everywhere: compartment-specific akt activation and function in receptor tyrosine kinase signaling. Front. Cel Dev. Biol. 7, 70. 10.3389/fcell.2019.00070 PMC6509475 31131274

[B96] Sun J. Zhang X. Wang C. Teng Z. Li Y. (2017). Curcumin decreases hyperphosphorylation of tau by down-regulating caveolin-1/GSK-3β in N2a/APP695swe cells and APP/PS1 double transgenic Alzheimer’s disease mice. Am. J. Chin. Med. 45, 1667–1682. 10.1142/S0192415X17500902 29132216

[B97] Tan Y. Yin L. Sun Z. Shao S. Chen W. Man X. (2020). *Astragalus* polysaccharide exerts anti-Parkinson via activating the PI3K/AKT/mTOR pathway to increase cellular autophagy level *in vitro* . Int. J. Biol. Macromolecules 153, 349–356. 10.1016/j.ijbiomac.2020.02.282 32112840

[B98] Thorpe L. M. Yuzugullu H. Zhao J. J. (2015). PI3K in cancer: divergent roles of isoforms, modes of activation and therapeutic targeting. Nat. Rev. Cancer 15 (1), 7–24. 10.1038/nrc3860 25533673 PMC4384662

[B99] Vaz M. Silvestre S. (2020). Alzheimer’s disease: recent treatment strategies. Eur. J. Pharmacol. 887, 173554. 10.1016/j.ejphar.2020.173554 32941929

[B100] Voulgaropoulou S. D. van Amelsvoort T. A. M. J. Prickaerts J. Vingerhoets C. (2019). The effect of curcumin on cognition in Alzheimer’s disease and healthy aging: a systematic review of pre-clinical and clinical studies. Brain Res. 1725, 146476. 10.1016/j.brainres.2019.146476 31560864

[B101] Wang G. Wang T. Zhang Y. Li F. Yu B. Kou J. (2019). Schizandrin protects against OGD/R-Induced neuronal injury by suppressing autophagy: involvement of the AMPK/mTOR pathway. Molecules 24 (19), 3624. 10.3390/molecules24193624 PMC6804185 31597329

[B102] Wang H. Dong X. Liu Z. Zhu S. Liu H. Fan W. (2018a). Resveratrol suppresses rotenone-induced neurotoxicity through activation of SIRT1/akt1 signaling pathway. Anat. Rec. 301 (6), 1115–1125. 10.1002/ar.23781 29350822

[B103] Wang H. Li Q. Sun S. Chen S. (2020). Neuroprotective effects of salidroside in a mouse model of Alzheimer’s disease. Cel. Mol. Neurobiol. 40 (7), 1133–1142. 10.1007/s10571-020-00801-w PMC11448871 32002777

[B104] Wang R. Yang S. Nie T. Zhu G. Feng D. Yang Q. (2017). Transcription factors: potential cell death markers in Parkinson’s disease. Neurosci. Bull. 33 (5), 552–560. 10.1007/s12264-017-0168-4 28791585 PMC5636736

[B105] Wang X. Pang L. Zhang Y. Xu J. Ding D. Yang T. (2018b). Lycium barbarum polysaccharide promotes nigrostriatal dopamine function by modulating PTEN/AKT/mTOR pathway in a methyl-4-phenyl-1,2,3,6-tetrahydropyridine (MPTP) murine model of Parkinson’s disease. Neurochem. Res. 43 (4), 938–947. 10.1007/s11064-018-2499-6 29594732

[B106] Wei Y. Zhou J. Yu H. Jin X. (2019). AKT phosphorylation sites of Ser473 and Thr308 regulate AKT degradation. Biosci. Biotechnol. Biochem. 83 (3), 429–435. 10.1080/09168451.2018.1549974 30488766

[B107] Wolkmer P. da Silva C. B. Paim F. C. Duarte M. M. M. F. Castro V. Palma H. E. (2013). Pre-treatment with curcumin modulates acetylcholinesterase activity and proinflammatory cytokines in rats infected with Trypanosoma evansi. Parasitol. Int. 62 (2), 144–149. 10.1016/j.parint.2012.11.004 23200738

[B108] Wu B. Chen Y. Huang J. Ning Y. Bian Q. Shan Y. (2012). Icariin improves cognitive deficits and activates quiescent neural stem cells in aging rats. J. Ethnopharmacology 142 (3), 746–753. 10.1016/j.jep.2012.05.056 22687254

[B109] Wu J. Qu J.-Q. Zhou Y.-J. Zhou Y.-J. Li Y.-Y. Huang N.-Q. (2020). Icariin improves cognitive deficits by reducing the deposition of β-amyloid peptide and inhibition of neurons apoptosis in SAMP8 mice. Neuroreport 31, 663. 10.1097/WNR.0000000000001466 32427716

[B110] Yan T. Sun Y. Gong G. Li Y. Fan K. Wu B. (2019). The neuroprotective effect of schisandrol A on 6-OHDA-induced PD mice may be related to PI3K/AKT and IKK/IκBα/NF-κB pathway. Exp. Gerontol. 128, 110743. 10.1016/j.exger.2019.110743 31629801

[B111] Yang W.-t. Zheng X.-w. Chen S. Shan C.-s. Xu Q.-q. Zhu J.-Z. (2017). Chinese herbal medicine for Alzheimer’s disease: clinical evidence and possible mechanism of neurogenesis. Biochem. Pharmacol. 141, 143–155. 10.1016/j.bcp.2017.07.002 28690138

[B112] Yang Z. Huang X. Lai W. Tang Y. Liu J. Wang Y. (2021). Synthesis and identification of a novel derivative of salidroside as a selective, competitive inhibitor of monoamine oxidase B with enhanced neuroprotective properties. Eur. J. Med. Chem. 209, 112935. 10.1016/j.ejmech.2020.112935 33097301

[B113] Yin J. Xiang C. Wang P. Yin Y. Hou Y. (2017). Biocompatible nanoemulsions based on hemp oil and less surfactants for oral delivery of baicalein with enhanced bioavailability. Int J Nanomedicine. Vol. 12, 2923. 10.2147/IJN.S131167 28435268 PMC5391827

[B114] Zarneshan S. N. Fakhri S. Farzaei M. H. Khan H. Saso L. (2020). Astaxanthin targets PI3K/Akt signaling pathway toward potential therapeutic applications. Food Chem. Toxicol. 145, 111714. 10.1016/j.fct.2020.111714 32871194

[B115] Zeng K. W. Wang X. M. Ko H. Kwon H. C. Cha J. W. Yang H. O. (2011). Hyperoside protects primary rat cortical neurons from neurotoxicity induced by amyloid β-protein via the PI3K/Akt/Bad/Bcl(XL)-regulated mitochondrial apoptotic pathway. Eur. J. Pharmacol. 672, 45. 10.1016/j.ejphar.2011.09.177 21978835

[B116] Zeng Z. Sarbassov D. D. Samudio I. J. Yee K. W. L. Munsell M. F. Ellen Jackson C. (2007). Rapamycin derivatives reduce mTORC2 signaling and inhibit AKT activation in AML. Blood 109 (8), 3509–3512. 10.1182/blood-2006-06-030833 17179228 PMC1852241

[B117] Zhai H. Kang Z. Zhang H. Ma J. Chen G. (2019). Baicalin attenuated substantia nigra neuronal apoptosis in Parkinson’s disease rats via the mTOR/AKT/GSK-3β pathway. J. Integr. Neurosci. 18 (4), 423–429. 10.31083/j.jin.2019.04.192 31912701

[B118] Zhang B. Wang Y. Li H. Xiong R. Zhao Z. Chu X. (2016a). Neuroprotective effects of salidroside through PI3K/Akt pathway activation in Alzheimer’s disease models. Drug Des. Devel. Ther. 10, 1335. 10.2147/DDDT.S99958 PMC4827895 27103787

[B119] Zhang C. Li C. Chen S. Li Z. Jia X. Wang K. (2017). Berberine protects against 6-OHDA-induced neurotoxicity in PC12 cells and zebrafish through hormetic mechanisms involving PI3K/AKT/Bcl-2 and Nrf2/HO-1 pathways. Redox Biol. 11 (C), 1–11. 10.1016/j.redox.2016.10.019 27835779 PMC5107737

[B120] Zhang L. Q. Sa F. Chong C. M. Wang Y. Zhou Z. Y. Chang R. C. C. (2015). Schisantherin A protects against 6-OHDA-induced dopaminergic neuron damage in zebrafish and cytotoxicity in SH-SY5Y cells through the ROS/NO and AKT/GSK3β pathways. J. Ethnopharmacology 170, 8–15. 10.1016/j.jep.2015.04.040 25934514

[B121] Zhang W. He H. Song H. Zhao J. Li T. Wu L. (2016b). Neuroprotective effects of salidroside in the MPTP mouse model of Parkinson’s disease: involvement of the PI3K/Akt/GSK3βPathway. Parkinson’s Dis. 2016, 1. 10.1155/2016/9450137 PMC5050371 27738547

[B122] Zhang X. Lai W. Ying X. Xu L. Chu K. Brown J. (2019a). Salidroside reduces inflammation and brain injury after permanent middle cerebral artery occlusion in rats by regulating PI3K/PKB/Nrf2/NFκB signaling rather than complement C3 activity. Inflammation 42 (5), 1830–1842. 10.1007/s10753-019-01045-7 31230155

[B123] Zhang X. Xiong J. Liu S. Wang L. Huang J. Liu L. (2014). Puerarin protects dopaminergic neurons in Parkinson’s disease models. Neuroscience 280, 88–98. 10.1016/j.neuroscience.2014.08.052 25218963

[B124] Zhang Y. Huang N. Chen M. Jin H. Nie J. Shi J. (2019b). Procyanidin protects against 6-hydroxydopamine-induced dopaminergic neuron damage via the regulation of the PI3K/Akt signalling pathway. Biomed. Pharmacother. 114, 108789. 10.1016/j.biopha.2019.108789 30925459

[B125] Zhang Y. Zhang L.-H. Chen X. Zhang N. Li G. (2018). Piceatannol attenuates behavioral disorder and neurological deficits in aging mice via activating the Nrf2 pathway. . Food Funct. 9(1), 371–378. 10.1039/c7fo01511a 29214257

[B126] Zhang Z. Cui W. Li G. Yuan S. Xu D. Hoi M. P. M. (2012). Baicalein protects against 6-OHDA-induced neurotoxicity through activation of keap1/nrf2/HO-1 and involving PKCα and PI3K/AKT signaling pathways. J. Agric. Food Chem. 60 (33), 8171–8182. 10.1021/jf301511m 22838648

[B127] Zhao M. Tang X. Gong D. Xia P. Wang F. Xu S. (2020). Bungeanum improves cognitive dysfunction and neurological deficits in D-galactose-induced aging mice via activating PI3K/Akt/Nrf2 signaling pathway. Front. Pharmacol. 11, 71. 10.3389/fphar.2020.00071 32158388 PMC7052015

[B128] Zhao W.-Z. Wang H.-T. Huang H.-J. Lo Y.-L. Lin A. M.-Y. (2017). Neuroprotective effects of baicalein on acrolein-induced neurotoxicity in the nigrostriatal dopaminergic system of rat brain. Mol. Neurobiol. 55 (1), 130–137. 10.1007/s12035-017-0725-x 28866823

[B129] Zheng M. Liu C. Fan Y. Yan P. Shi D. Zhang Y. (2017). Neuroprotection by Paeoniflorin in the MPTP mouse model of Parkinson’s disease. Neuropharmacology 116, 412–420. 10.1016/j.neuropharm.2017.01.009 28093210

[B130] Zhi Y. Jin Y. Pan L. Zhang A. Lin F. (2019). Schisandrin A ameliorates MPTP-induced Parkinson’s disease in a mouse model via regulation of brain autophagy. Arch. Pharm. Res. 42(11), 1012–1020. 10.1007/s12272-019-01186-1 31552591

[B131] Zhuang W. Li T. Wang C. Shi X. Li Y. Zhang S. (2018). Berberine exerts antioxidant effects via protection of spiral ganglion cells against cytomegalovirus-induced apoptosis. Free Radic. Biol. Med. 121, 127–135. 10.1016/j.freeradbiomed.2018.04.575 29715550

[B132] Zou X. Feng X. Fu Y. Zheng Y. Ma M. Wang C. (2020). Icariin attenuates amyloid-β (Aβ)-Induced neuronal insulin resistance through PTEN downregulation. Front. Pharmacol. 11. 10.3389/fphar.2020.00880 PMC7296100 32581820

[B133] Zuardi A. Crippa J. Hallak J. Pinto J. Chagas M. Rodrigues G. (2009). Cannabidiol for the treatment of psychosis in Parkinson’s disease. J. Psychopharmacol. 23 (8), 979–983. 10.1177/0269881108096519 18801821

