# Supplementary material for: PI3K/AKT Signal Pathway: A Target of Natural Products in the Prevention and Treatment of Alzheimer’s Disease and Parkinson’s Disease
Source: Front Pharmacol. 2021 Apr 15;12:648636. doi: 10.3389/fphar.2021.648636 (PMC8082498; doi:10.3389/fphar.2021.648636)
Supplement: Supplementary file 1 [file DataSheet1.pdf]

Supplementary Material

Supplementary Figures

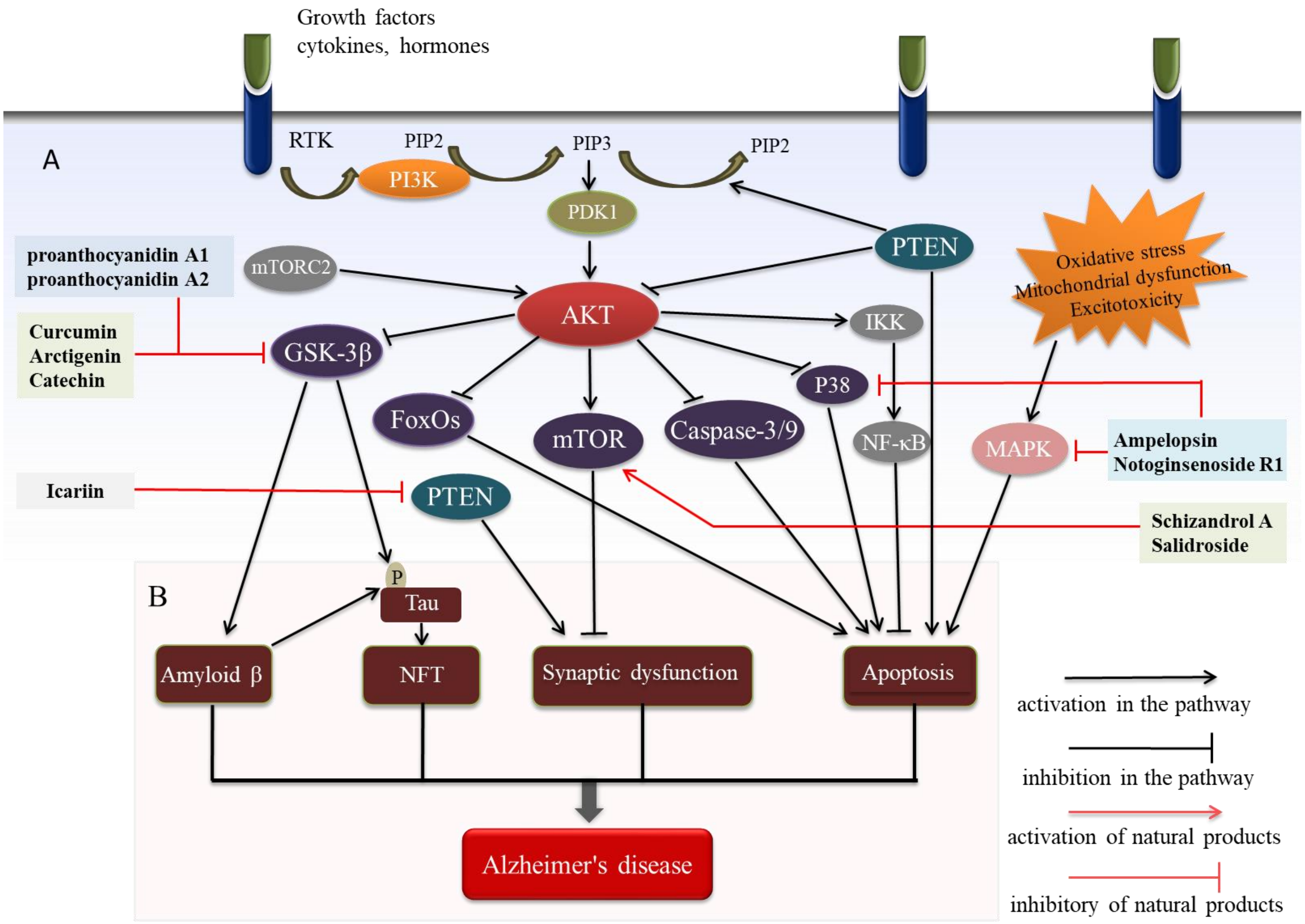

Supplementary Figure 1. Schematic depiction of PI3K/AKT signaling in AD pathogenesis is shown. Examples of natural products known to act on the PI3K/AKT signaling pathway to alleviate AD symptoms are also shown. (A) PI3K/AKT signaling pathway; (B) molecular pathogenesis of AD. According to (A) and (B), AKT is activated and regulated by receptor tyrosine kinase (RTK) via PI3K pathway, phosphorylates or inhibits its downstream target substrate, and regulates diverse biological functions, participating in the pathological regulation of AD. Various natural products slow the progression of AD through the AKT pathway. Curcumin and arctigenin inhibit production of Aβ and tau protein phosphorylation through PI3K/AKT/GSK-3β pathway. Catechin, proanthocyanidin A1 and proanthocyanidin A2 restrains hyperphosphorylated Tau protein by inhibiting GSK-3β. Icariin protects synapses by downregulating PTEN. Ampelopsin and notoginsenoside R1 alleviate neuronal apoptosis of AD by inhibiting p38 and MAPK phosphorylation. Schisandrol A and Salidroside inhibit autophagy via mTOR activation, improving

synaptic structure. Note that each drug may have more than one pathway of action, and some events have been omitted for clarity.

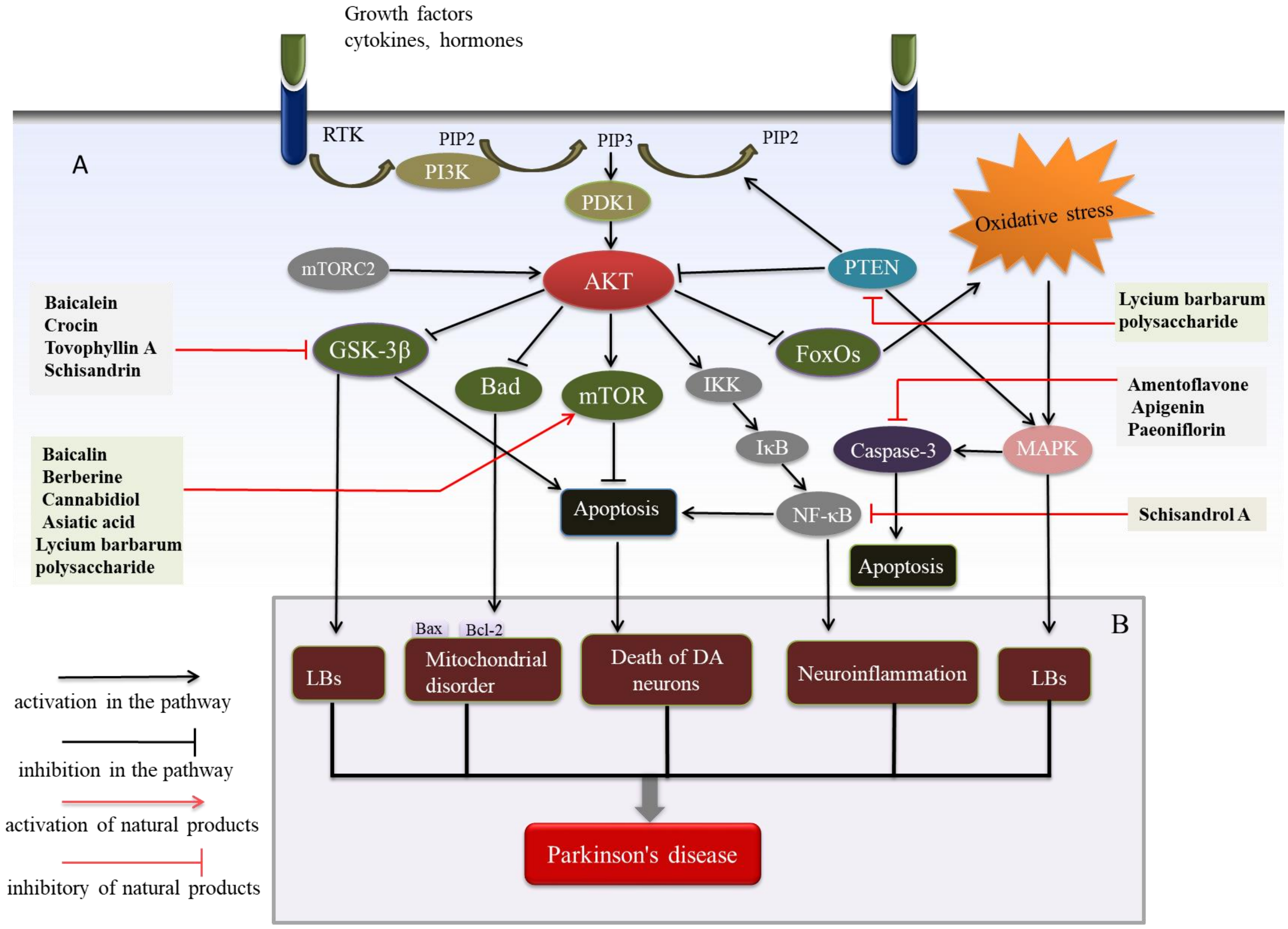

Supplementary Figure 2. Schematic depiction of PI3K/AKT signaling in PD pathogenesis is shown. Examples of natural products known to act on the PI3K/AKT signaling pathway to alleviate PD symptoms are also shown. (A) PI3K/AKT signaling pathway; (B) molecular pathogenesis of PD. According to (A) and (B), AKT is activated and regulated by receptor tyrosine kinase (RTK) via PI3K pathway, phosphorylates or inhibits its downstream target substrate, and regulates diverse biological functions, participating in the pathological regulation of PD. Various natural products slow the progression of AD through the AKT pathway. Baicalein, crocin, tovophyllin A and schisandrin act on PI3K/AKT/GSK-3β pathway, slow down the formation of LBs and inhibit nerve cell death. Baicalin, berberine, cannabidiol, asiatic acid and lycium barbarum polysaccharide prevent apoptosis via AKT/mTOR pathway. Lycium barbarum polysaccharide activates the AKT/mTOR pathway through inhibiting PTEN. Amentoflavone apigenin and paeoniflorin reduce caspase-3 activity, improving the lesioned neurobehavio. Schisandrol A alleviates the nerve injury and neuroinflammation by inhibiting the NF-κB pathway. Note that each drug may have more than one pathway of action, and some events have been omitted for clarity.
